# The Crude Glycerol Challenge: Purification Technologies and Possible Valorization Pathways in the Biodiesel Industry

**DOI:** 10.3390/molecules31111841

**Published:** 2026-05-27

**Authors:** Consolato Rosmini, Yavor Mitrev, Momtchil Dimitrov

**Affiliations:** Institute of Organic Chemistry with Centre of Phytochemistry, Bulgarian Academy of Sciences, Acad. G. Bonchev Street, Bl. 9, 1113 Sofia, Bulgaria; yavor.mitrev@orgchm.bas.bg (Y.M.); momtchil.dimitrov@orgchm.bas.bg (M.D.)

**Keywords:** glycerol valorization, crude glycerol purification, biorefinery processes, sustainable catalysis

## Abstract

The rapid expansion of the biodiesel industry has led to a significant surplus of crude glycerol, creating both a challenge and an opportunity for its sustainable valorization. The aim of this review is to provide a comprehensive overview of glycerol upgrading strategies that integrate upstream purification methods with downstream catalytic conversion pathways. Conventional and emerging purification technologies are critically discussed, with particular attention to their efficiency, cost, and compatibility with catalytic processes. Major catalytic routes such as acetalization, oligomerization, carbonation, hydrogenolysis, oxidation, etherification, esterification and steam reforming are systematically analyzed in terms of reaction mechanisms, catalyst design, and process conditions. Although high conversions and selectivities are often achieved under optimized conditions, key limitations such as catalyst deactivation, equilibrium constraints, feedstock impurities, and scalability issues remain significant barriers to industrial implementation. The review highlights the strong interdependence between glycerol purity, catalytic performance, and process design, emphasizing the need for integrated approaches. Recent advances in multifunctional catalysts, reactor engineering, and process intensification are discussed as promising strategies to overcome current challenges. Overall, this work provides a critical perspective on the state of the art and identifies future directions toward the development of efficient, scalable, and sustainable glycerol-based biorefinery processes.

## 1. Introduction

**Figure 1:**
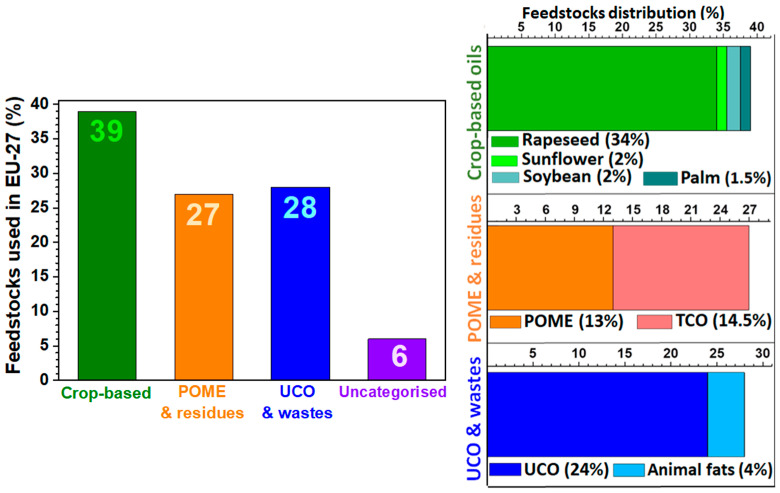
Feedstock distribution for FAME and HVO production in the EU-27 (**Left panel**) and detailed breakdown by feedstock category (**right panel**). Data extrapolated and adapted from the EBB report 2024–2025 [4].

As a result of the climate crisis and the various decarbonization plans adopted by most of the world countries, since 2002 there has been a sharp increase in international biodiesel production with the aim of mitigating the use of current fossil fuels and reducing the anthropogenic carbon footprint. Driven by the increasingly stringent carbon emission reduction policies, the global biodiesel market reached a value of approximately USD 40.3 billion in 2025 and is projected to grow to nearly USD 67.4 billion by 2034 [1]. Such an expansion reflects the strategic role of biodiesel in national decarbonization plans and renewable fuel mandates worldwide. In October 2024, Brazil enacted the “Fuel of the Future” law, which established a gradual increase in biodiesel blending mandates by 1% per year starting in 2025, with the objective of reaching a total of 20% biodiesel blend (B20) by 2035. In the same year, Brazil ranked among the world’s leading biodiesel producers, with an output of 8.9 billion liters (approximately 7.8 Mt). Indonesia, another key global producer, completed the first year of its B35 blending mandate in 2024, achieving a total biodiesel production of 13 billion liters (11.4 Mt), of which 12.6 billion liters were allocated to domestic consumption [2]. In contrast, the United States experienced a slight production decline of about 1% compared to 2023, reaching 6.3 billion liters (5.5 Mt). Meanwhile, the European Union continues to represent the largest market for bio-based diesel (BBD), both in terms of production and consumption [2]. From a European perspective, in the context of the directives otherwise known as the “European Green Deal” (formalized in the document (EU) 2021/1119) [3], it was established the objective of the Union to reach a climate neutrality by 2050 and an intermediate target of a reduction of net greenhouse gas emissions by at least 55% compared to 1990 levels by 2030. However, even before the “European Green Deal”, the EU’s legislation had been paying particular attention to bio-based components added to diesel fuel; in fact, during 1998, the Directive 98/70/EC set the amount of bio-derived compounds in the fuel to a maximum of 7%, naming them biodiesel “B7”. At present, the most recent European Parliament directive ((EU) 2023/2413) [3] has established that the majority of biodiesel currently consumed in EU countries is class B7, and has formalized a further increase in the biogenic component, mostly fatty acid methyl esters (FAMEs), to 10% (B10) of fuel by 2030, thus prospecting to a further increase in the consumption of these bio-based additives in the near future. According to the statistical report of the year 2024–2025 [4] from the European Biodiesel Board (EBB), whose members represent around 70% of the European biodiesel producers, in 2024, there was a slight increase in production, with approximately 9.46 Mt of European-produced biodiesel compared to the previous year (9.33 Mt) [4]. Of these, approximately 75% consists of FAMEs, while approximately 25% consists of hydrotreated vegetable oils (HVOs) and hydrotreated esters and fatty acids (HEFAs). To be slightly more precise, the report also shows that, in addition to the constant dominance of FAMEs in the European market compared to HVOs and HEFAs, the actual yearly market demand exceeds the EU-27’s production supply by approximately 29%, with 12.1 Mt actually required to meet EU demand. The countries that contribute most to this are: France with 2.9 Mt/y2024, Germany 2.7 with Mt/y2024, Spain with 1.8 Mt/y2024, and Italy with 1.5 Mt/y2024, while the rest of the European Union countries have cumulatively produced 6.8 Mt/y2024. However, the limited FAME production within the EU-27 is mainly constrained by the availability of lipid feedstocks, as FAME synthesis relies predominantly on the acid- or base-catalyzed transesterification of vegetable and/or animal triglycerides with methanol. At this stage (see Figure 1), the 39% of these esters are still produced from crop-based feedstock such as rapeseed oil, sunflower oil, soybean oil and palm oil, with rapeseed oil accounting for the largest contribution (34% of the total used feedstock) due to its established agricultural infrastructure and lower regulatory constraints compared to other vegetable oils [5]. In fact, the extensive use of edible vegetable oils is increasingly restricted by European policies in order to mitigate fuel-versus-food competition and indirect land use change (ILUC) [6]. In view of these objective problems, the expansion of crop-based biodiesel production in the EU-27 is therefore structurally constrained. The remaining share of FAME production relies predominantly on waste-derived feedstock, particularly used cooking oil (UCO), which accounts for 24% of the global raw material used, but its availability is inherently limited by collection efficiency, logistics, and society participation in the waste management schemes. Additional waste-based feedstock includes palm oil mill effluent (POME), contributing approximately 13% of total biodiesel production, along with other residues such as technical corn oil (TCO), category 3 animal fats and palm fatty acid distillates (PFADs), which together play a key role in supplementing the existing feedstock portfolio for FAME and also HVO synthesis, even if in very modest quantities (Figure 1).

The progressive global expansion of biodiesel production inevitably generates significant amounts of crude glycerol as a direct industrial byproduct of the transesterification process. Glycerol itself is a highly versatile molecule that currently holds an established market value due to its wide range of industrial applications. In addition, it represents a promising platform molecule that can be converted through various catalytic pathways into a variety of value-added chemicals and fine chemicals. However, the effective valorization of crude glycerol faces several challenges. These issues already arise at the purification stage, where the complex composition of the crude stream complicates its upgrading, and continue with technological and catalytic limitations that still hinder the full exploitation of its potential. This review aims to provide an overview of the benefits and limitations associated with this value chain, outlining the broader background of this challenge. Particular attention is devoted to the most recent developments in the heterocatalytic valorization of refined glycerol, describing the current limitations in the transformation routes of this valuable molecule.

## 2. Production of FAME and Glycerol via Methanol Transesterification

**Figure 2:**
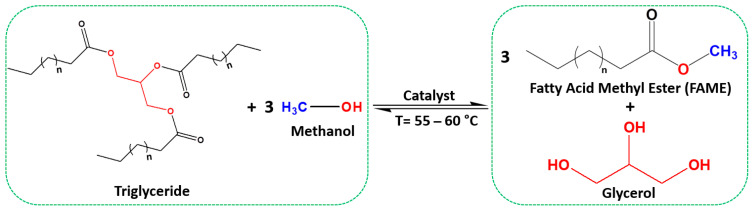
Triglyceride transesterification to produce FAME and glycerol.

The transesterification reaction is undoubtedly the most widely used method for producing biodiesel from vegetable triglycerides (TAGs) at an industrial scale nowadays [7]. In this reaction, which is generally catalyzed homogeneously, one mole of TAGs reacts with three moles of a primary chain alcohol such as methanol (or ethanol for the fatty acid ethyl esters) to produce three moles of methyl esters and one mole of glycerol, as shown in Figure 2. The reaction generally occurs at relatively low temperatures (55–60 °C), just below the boiling point of methanol (65 °C). Furthermore, since the transesterification reaction is reversible, an excess of alcohol is generally used to maximize yields. Although esterification reactions can be catalyzed by both acids and strong bases, in the case of biodiesel production, the choice of the catalytic environment depends specifically on the type of feedstock used. In fact, the presence of water and free fatty acids (FFAs) severely limits the production of esters. In the case of alkali-catalyzed transesterification using NaOH or KOH, the presence of FFA would result in the formation of soaps, which would decrease the yields of the process [8]. As mentioned in the previous paragraph, more than 39% of the biodiesel produced in Europe comes from untreated vegetable oils with a high content of triglycerides. For this reason, on a large scale, the biodiesel currently produced from this feedstock is obtained via alkali-catalyzed one-step transesterification to maximize yields while minimizing production costs.

However, as already mentioned in the introduction, the percentage of biodiesel produced from used cooking oils (UCOs) or similar types of waste currently accounts for only 28% of the feedstock used in the EU-27. Unlike untreated vegetable oils, the FFA content in UCO could vary from 0.5 up to 15 wt.%. In fact, during the cooking process of these oils, there can be at least three chemical–physical processes (oxidation, hydrolysis and thermolysis [8]) that damage the triglyceride component, necessary for the formation of FAMEs, in favor of the more problematic, albeit still usable, fatty acid fraction. These fractions, some examples of which are listed in Table 1, make the alkali-catalyzed transesterification process less efficient due to the almost immediate formation of soaps [8,9]. In addition to the competitive saponification process, UCOs also have less advantageous chemical–physical characteristics than untreated vegetable oils, such as higher viscosity, higher surface tension and higher relative acidity, which considerably increase the mass transfer limitations, thus affecting the reaction yields [8,9,10].

To overcome this problem, the most commonly used approach is a double-step transesterification process (TDSP). In this process, UCOs are first treated with strong acids, such as H_2_SO_4_, in order to esterify the FFAs first, reducing them to less than 1 wt.%. Subsequently, in the second step, the reactive mixture is treated with highly alkaline solutions to transesterify the remaining TAG components into FAMEs. From a yield perspective, both processes can be considered advantageous but certainly improvable [15]. Some examples starting directly from non-edible vegetable oils report yields above 90%, as in the case of Almady et al. [16]. In their study, the authors also demonstrated the possible use of high-voltage electrodes in the reactor for the simultaneous separation of the glycerol fraction from that of biodiesel, using Pongamia and Jatropha seed oils, respectively. With this technique, they achieved conversions of over 93% at a temperature of 55 °C in one hour of reaction, with a methanol/oil ratio = 5 (*v*/*v*) and approximately 1% *w*/*w* NaOH as a catalyst. Chamara et al. reported a rather efficient method for the valorization of POME through preventive precipitation of FFAs with a sequence of centrifugation and alkaline treatment, obtaining a yield of 71% with a methanol/oil ratio = 8 (*v*/*v*) but managing to reuse about 96% of the effluent body [9]. Bajwa et al. proposed a design of experiment focused on the use of response surface methodology (RSM) and optimized through the use of artificial neural networks (ANNs). By varying parameters such as reaction speed, reaction time and methanol/oil ratio for microwave-induced transesterification of sesame seed oil, the authors were able to optimize biodiesel yield to 94% using a mixing rate of 350 rpm, approximately 1% *w*/*w* catalyst (KOH), methanol/oil ratio = 10 (*v*/*v*) and a reaction time of only 3 min [17]. Given the high efficiency and industrial maturity of the transesterification process, biodiesel production has become a well-established technology. However, as shown in Figure 2, for every ten parts of biodiesel produced, approximately one part of crude glycerol is generated as a byproduct. This has led to a significant surplus of low-purity glycerol, raising concerns about its sustainable management and valorization. As a result, glycerol utilization has emerged as a critical challenge and opportunity within the biodiesel value chain.

## 3. Crude Glycerol as an Unavoidable Byproduct: Composition and Challenges

**Figure 3:**
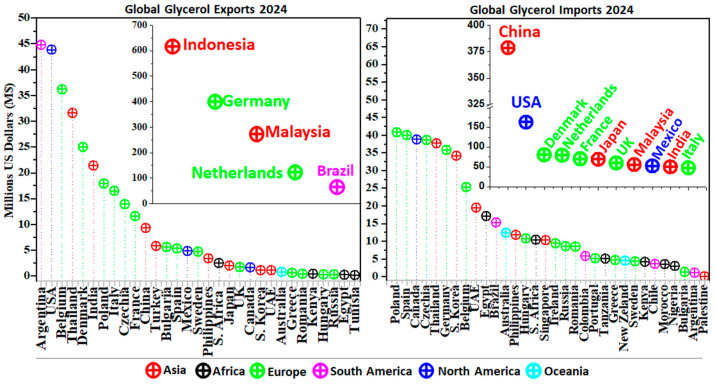
Global imports and exports of glycerol (including synthetic glycerol) in 2024. Insets: top glycerol importing and exporting countries. Data acquired and reprocessed from the latest OEC report from 2024 [21].

As previously discussed, the transesterification reaction of triglycerides with methanol, in addition to FAMEs used as biodiesel, inevitably also produces a glycerol component. According to the literature [18,19], glycerol accounts for approximately 10 wt.% of the total mass of biodiesel produced, depending on the yield of the process used. Calculating on the quantities of biodiesel produced in the EU-27, previously discussed in the UBB report (2024–2025), the glycerol component produced from 9.46 Mt is therefore in the range of 1–1.2 Mt of crude glycerol generated each year in the European Union. As for other major biodiesel producing countries such as the USA, Brazil and Indonesia, the quantities of crude glycerol produced can be estimated to be approximately 0.61, 0.87 and 1.27 Mt, respectively. It is therefore estimated that approximately 4.1 million tons would be produced globally each year, of which, according to Haider et al., 680 Kt can already be considered heavily compromised for hypothetical reuse due to the high concentration of impurities [20]. Pure glycerol, whether bio-based or synthetic (the latter currently accounting for approximately 25% of the global market), has a significant import–export market value. The latest report from the Observatory of Economic Complexity (OEC, 2024 [21]) states that global trade in this product is worth $1.79 billion dollars and that it ranks 1527th in terms of economic importance among approximately 4637 traded products, although 2024 saw a 11.1% decline in trade compared to the previous years ($2.02 billion traded in 2023 and $3.3 billion in 2022). Figure 3 clearly shows that this is a product which, once purified, is traded on virtually every inhabited continent. The largest exporters are Indonesia ($616 million), Germany ($396 million), Malaysia ($263 million) and Australia ($80.3 million). The largest importers are China ($379 million), the United States ($163 million), the Netherlands ($79.3 million), Denmark ($80.6 million) and France ($70.7 million) [21]. Looking at Figure 3, it can be noticed that the largest producers of massive quantities of biodiesel and, therefore, crude glycerol, such as the USA and China, are unable to meet their domestic demand and are, therefore, the top importers of this product. Another reason, not necessarily in contrast with the first, is that these countries would prefer to import purified or semi-purified glycerol from other biodiesel-producing countries with low domestic demand for this good in order to reduce purification costs.

Although glycerol is a marketable product particularly present in our daily lives, especially in the cosmetics [22], food [23], tobacco [24] and pharmaceutical [25] industries, biodiesel producers face serious economic constraints in the large-scale use of the vast quantities of crude glycerol they produce, due to the prohibitive costs of purification. In order to be marketed, glycerol must comply with the purity criteria imposed by the regulatory authorities. In the US, for example, the Food and Drug Administration (FDA) regulates and monitors reagents for pharmaceutical and food use, continuously updating the United States Pharmacopeia (USP) and the Food Chemicals Code (FCC). Specifically for glycerol, the USP monograph “USP 32-NF 27 2nd Supplement” states that the content of ethylene glycol and diethylene glycol declared dangerous for human consumption is not permissible in quantities exceeding 0.1 wt.%, respectively, limiting the applications of synthetic glycerol. It goes without saying that there are, therefore, at least three different grades of pure glycerol available for sale in the US [26]:

(1) Grade I (technical grade 99.5%): A synthetic product used as a chemical reagent but prohibited for pharmaceutical and food use.
(2) Grade II (USP grade 96–99.5%): Produced by refining crude glycerol derived from plant and/or animal sources, applicable for the pharmaceutical and food industries.
(3) Grade III (Kosher or USP/FCC grade 99.5%): Produced by refining crude glycerol derived from plant oils, but not animals, suitable for kosher foods and drinks (or similar).

Similar concerns regarding the use of this product are found within the European Union, where products such as glycerol are regulated and monitored by the European Directorate for the Quality of Medicines & Health Care (EDQM) and the European Food Safety Authority (EFSA). For example, in the EU-27 countries, glycerol is permitted for use as a food excipient (E422) with a purity of not less than 98% and with well-defined and mandatory chemical and physical characteristics, most recently revised in 2017 [27], for both member countries that produce it and for the imported products. Being technically a waste product, the final price of purified glycerol depends mainly on the processes used to extract and purify this molecule from the “crude glycerol” fraction coming directly from biodiesel production plants. However, this fraction differs significantly from refined glycerin in color, odor, and chemical–physical properties, making it not directly usable for common purposes. In particular, the glycerol content, as well as the nature and distribution of impurities, are strongly influenced by the efficiency of the transesterification process and the origin of the triglyceride raw material.

As summarized in Table 2, variations in raw material composition and transesterification operating conditions result in glycerol content ranging from 33 wt.% [28] to 81.7 wt.% [29]. The crude glycerol stream contains significant amounts of contaminants, mainly classified as MONG (Matter Organic Non-Glycerol), generally consisting of a variable distribution of free fatty acids (FFAs), fatty acid methyl esters (FAMEs), partial glycerides (mono-, di-, and triglycerides), and saponified fatty acids, whose total concentration is generally higher when there is low transesterification efficiency in the processes.

Residual methanol and water concentrations can also vary significantly from one crude glycerol batch to another, depending on the alcohol to oil molar ratio used, which in turn is selected based on the characteristics of the feedstock and the specific configuration of the plant. As a further consequence, the pH of the crude product may also differ substantially, depending on the combination of acid and alkali treatments applied during the transesterification process, as described in Section 2. Given the high composition variability of crude glycerol, it is particularly difficult to establish a standardized industrial procedure for its purification to the quantities and purity levels required by the market. This is particularly true when the bulk raw material comes from a variety of waste sources from different production plants, each using a different feedstock, as the application of customized physicochemical purification methods would inevitably increase both energy consumption and overall process costs [18].

## 4. Conventional Glycerol Purification Routes and Limitations

**Figure 4:**
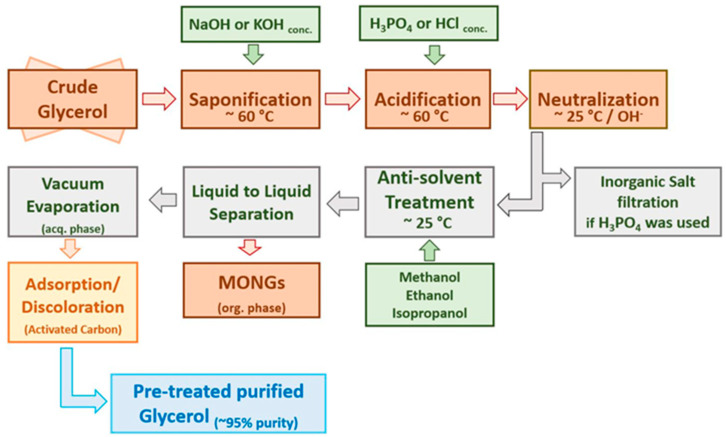
Illustrative flow chart of crude glycerol pretreatment and purification steps.

The purification and rectification processes of crude glycerol consist of a series of chemical–physical reactions that allow the sequential separation of molecules contained as impurities within the glycerol component in order to obtain the highest yield and degree of purity of the latter. At first, since the crude is a byproduct of the biodiesel industry and methanol is typically used in excess during the transesterification, the primary treatment applied to this waste phase (as well as to the biodiesel phase) is the recovery of the unreacted alcohol for its reuse. This step is generally performed by distillation at approximately 65–70 °C at atmospheric or reduced pressure, depending on the plant configuration. Alternatively, methanol can be recovered by flash evaporation or vacuum stripping units [18] integrated into the separation section downstream of the biodiesel production process. Once the most volatile component has been removed, the crude glycerol fraction generally undergoes a sequential process of alkaline saponification, acidification, and neutralization in order to reduce all MONG components by first converting them into soaps and then into FFAs emulsified in a mixture of water, glycerol, and inorganic salts [32]. Due to their poor solubility in water, free fatty acids can be efficiently recovered by liquid–liquid extraction using organic solvents such as 2-propanol [32], petroleum ether, n-butanol [33], or methanol [34], after which the organic phase is physically separated from the aqueous glycerol solution. The latter will be further subjected to distillation in order to eliminate traces of organic solvent and water, thus concentrating the glycerol, which will then undergo a further adsorption phase using activated carbon in order to remove any traces of chromophore or odorous molecules [34]. A conceptual representation of the multistep process is shown in Figure 4.

Despite its robustness, this chemical purification sequence is labor-intensive, poorly standardized, and associated with high operational costs. Furthermore, it generally provides limited glycerol purities (≤95 wt.%) and moderate recoveries, strongly dependent on feedstock variability. Consequently, it is currently implemented mainly as a bulk pretreatment stage prior to more selective and scalable separation technologies such as vacuum distillation [35], membrane-based processes [36] and ion exchange [19].

### 4.1. Vacuum Distillation (VD)

Due to the thermal sensitivity of glycerol and its contaminants at high temperatures, distillation at ambient pressure is not feasible for purifying any type of crude glycerol derivative, given that the boiling point of glycerol itself is 290 °C. For this reason, vacuum distillation is the only plausible thermo-physical separation method and currently the most established technology for the purification of glycerol for commercial purposes [35]. Selected due to its robust technological background, this method nevertheless faces several challenges when applied to crude glycerol, which can affect both its technical performance and economic feasibility. First, the feed must be pretreated using the methods previously described, since processing raw glycerol directly as a whole is not particularly feasible, as the high presence of MONG and inorganic salts could cause column bottom blockage. Furthermore, the use of raw material with a high salt concentration (2–5 wt.%) also requires the use of a scraped-wall evaporator [37] to prevent salt accumulation on the inner walls of the separator. Careful control of the initial pH of the pretreated glycerol solution is critical. Under strongly alkaline conditions (high pH) in the presence of NaOH and at temperatures near 200 °C, glycerol is prone to polymerization. Conversely, under acidic conditions (pH < 3), the triol can undergo dehydration, leading to the formation of acrolein [19]. Since this process uses a high vacuum (10–30 mbar) [38] in industrial applications, the water content inside the feedstock must not exceed 10–15 wt.% in order not to affect the stability of the vacuum inside the evaporator, generating additional energy costs. Although it remains the most widely used process industrially at present, the experimental academic research on the topic, especially in the last 15 years, appears to be rather scarce. The work of Yong et al. ([39] from 2001) is pivotal in this sense, having distilled 1 kg of crude glycerol with a high salt and MONG content, recovering approximately 141.8 g of glycerol with a purity of approximately 96.6% by distillation at a temperature range of 120–126 °C and a pressure of 0.4 to 0.04 mbar. Although vacuum distillation remains the most established industrial technique for crude glycerol purification, it is intrinsically energy-intensive and associated with high operational and maintenance costs. Moreover, the presence of salts and MONGs often leads to fouling, thermal degradation, and reduced glycerol recovery when insufficient pretreatment is applied. These limitations significantly affect the economic sustainability of the process, especially for highly variable crude glycerol streams.

### 4.2. Membrane Purification (MP)

**Figure 5:**
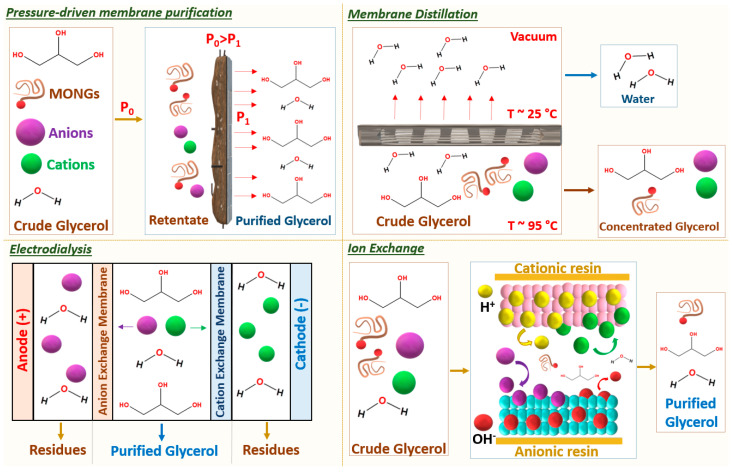
Schematic representation of the functioning of some glycerol purification methods.

Membrane separation technology represents an emerging approach in crude glycerol purification (Figure 5). Under isothermal conditions, the driving force of these membrane-assisted methods involves mostly the differences in hydrostatic pressure or electrical potential. Such parameters, in combination with the specific membrane materials employed, determine the major classes of membrane separation technologies, including microfiltration (MF), ultrafiltration (UF), nanofiltration (NF), reverse osmosis (RO), and electrodialysis (ED). With respect to the operating principle of so-called pressure-driven separation membranes, crude glycerol is pressurized against a semi-permeable membrane, which is typically polymeric, ceramic, or based on hybrid matrix materials extensively detailed by Govindaraju et al. [36]. Through the optimization of key operating parameters, such as temperature, transmembrane pressure (TMP) and nominal molecular weight cut-off (MWCO), the membrane enables the selective passage of the permeate, consisting mainly of the glycerol–water mixture, while blocking the so-called “retentate”, which in this case is primarily composed of MONGs and inorganic salts [40]. Under these conditions, both the physicochemical properties of the membrane and the applied TMP difference play a crucial role in determining process efficiency. Gomes et al. [41] demonstrated that tangential MF and UF filtration of crude glycerol using ceramic membranes (α-Al_2_O_3_/TiO_2_) with nominal pore sizes of 5 kDa, 20 kDa, 0.05 μm, and 0.2 μm was strongly influenced by transmembrane pressure (from 1 to 3 bar used), temperature, and membrane pore size. Increasing the applied pressure, pore diameter, and temperature led to higher permeate fluxes, while the addition of acidified water further enhanced both flux and glycerol recovery. The highest glycerol purity (91.1%) was achieved with the multichannel 5 kDa membrane at 3 bar and 60 °C. Tajziehchi et al. [42] used a commercial polyvinylidene fluoride (PVDF) ultrafiltration membrane with a 100 kDa MWCO employed to treat crude biodiesel. Using response surface methodology (RSM), varying temperatures, TMP and water addition on the removal of free glycerol, diglycerides, and triglycerides, as well as on permeate flux, the authors found that simultaneous reduction of all targeted impurities to levels compliant with international standards could be achieved at pressures below 2 bar and temperatures beneath 30 °C. However, these conditions resulted in a relatively low permeate flux (~20 kg m^−2^·h^−1^). Despite the potential of membrane-based technologies as environmentally friendly alternatives to conventional biodiesel purification, their large-scale implementation remains limited by several intrinsic challenges. Fouling is the dominant issue [43], mainly caused by glycerol agglomeration in the presence of residual alcohols, soaps, and catalysts, leading to pore blocking, cake-layer formation, concentration polarization, and rapid permeate flux decline [44]. Membrane material selection further constrains process optimization; in fact, polymeric membranes often exhibit poor mechanical and chemical resistance and short lifetimes, while ceramic membranes, although more robust, suffer from high costs and reduced permeability at high selectivity. Moreover, membrane performance is strongly dependent on feed composition, as soap formation can deactivate the active sites and decrease glycerol retention by disrupting agglomerate structures. Finally, frequent cleaning requirements, limited removal of residual MONGs and salts, and the lack of mature scale-up strategies collectively reduce the economic feasibility and long-term sustainability of membrane-based biodiesel purification.

### 4.3. Membrane Distillation (MD)

Another emerging technique is based on non-isothermal membrane separation processes (Figure 5). The main applications of this technology are primarily the concentration of aqueous glycerol mixtures, thus assisting vacuum distillation processes in order to minimize energy losses resulting from the evaporation process [36]. As mentioned above, the latter requires a high absolute energy input, which, despite better use of the applied thermal energy, with a gain–output ratio (GOR) ranging between 3 and 5 [45], makes it an energy-intensive process. From a purely historical point of view, the first MD method used is Direct Contact Membrane Distillation (DCMD), which consists of a hydrophobic porous polymer membrane that separates the feed zone, generally at a higher temperature (90–100 °C), from the diametrically opposite zone in which a cooling liquid flows (25 °C). When it comes into contact with the membrane, the polar feed is retained by the hydrophobic membrane, but the water contained in the feed evaporates and, passing through the membrane, condenses on contact with the cold area of the cooling liquid, thus making the temperature difference the main driving force in this method [36]. However, although DCMD has energy-saving advantages, its original configuration still has a GOR of just under 1, which is not considered advantageous due to the energy required to preheat the feed, provide the latent heat of evaporation of the water, and compensate for the enormous heat losses absorbed and dissipated by the membrane [45]. For this reason, massive research efforts have been made to implement its configuration and reduce energy costs. Some examples are Vacuum Membrane Distillation (VMD) [46], Sweeping Gas Membrane Distillation (SGMD) [47], and Continuous-Effect Membrane Distillation (CEMD). Zhang et al. [45], for example, implemented a CEMD using two different types of hollow fiber-based Air Gap Membrane Distillation (AGMD) modules in order to achieve in situ recovery of the latent heat of the distillate on a solution with a high concentration of glycerol in water starting from 10 g/L and concentrating it up to 400 g/L with a rejection efficiency greater than 99.9% and reaching a maximum GOR of 16.2. It is interesting to note that as the concentration of glycerol increased, resulting in an increase in the viscosity of the solution (after approximately 300 g/L), the GOR underwent a sudden decrease to approximately 5.3, which is still comparable to that of a traditional six-effect evaporator. At present, this technique can be considered primarily as an auxiliary method for concentrating clean aqueous glycerol solutions. Its application to unrefined crude glycerol would not only lead to membrane fouling due to MONGs but would also concentrate these impurities along with the glycerol, failing to address the underlying purification challenge.

### 4.4. Electrodialysis (ED)

Electrodialysis is a technique that removes ionic species from a solution by applying a voltage, creating an electric field that drives ions through selective ion-exchange membranes, effectively separating them from the mixture (Figure 5). In the context of glycerol purification, this method has been applied mainly on a laboratory scale to desalinate aqueous solutions of synthetic glycerol containing different concentrations of dissolved NaCl using commercial membranes. De Schepper et al. [48], for example, desalinated synthetic NaCl–glycerol–water mixtures at varying concentrations of the three components from 0.06 M to 0.6 M, investigating seven different ion-exchange membrane pairs (anionic–cationic) from different manufacturers, achieving 90% desalination after less than 3 h. Rozhdestvenskaya et al. [49] developed organo-inorganic ion-exchange membranes by modifying polymeric commercial membranes (CMI 7000 and AMI 7001) with hydrated zirconium dioxide. The latest modified membranes have proven to be effective for electrodialysis of glycerol–water mixtures containing organic impurities (8 wt.%) in the presence of NaCl (100–1500 mol/m^3^), showing higher resistance to fouling than unmodified membranes and achieving current efficiencies of 95–98% over extended operation. About 90% desalination was obtained after 70 h, while organic impurities remained in solution, indicating that electrodialysis is best suited as a preliminary desalination step. The study conducted by Attarbachi et al. [50] evaluated electrodialysis under realistic crude glycerol impurity conditions. In that work, two industrial glycerol feedstocks were desalinized after physicochemical pretreatment and dilution with deionized water to 50 wt.% (with salt/ash contents ranging from 5 to 15%). By first optimizing the operating parameters using synthetic glycerol solutions (ash content < 5 wt.% at an applied voltage of 16.28 V), the authors reported a glycerol recovery of approximately 71.4% within 136 min, with a specific energy consumption of about 4.51 MWh·m^−3^. When the process was applied to pretreated industrial crude glycerol, the average glycerol recovery decreased to around 62.2% after 282.7 min, while the specific energy consumption increased to approximately 8.85 MWh·m^−3^. As expected, the presence of MONGs and diverse salt species in the industrial samples led to competitive transport phenomena and membrane fouling. In particular, a noticeable yellowish deposit attributed to MONGs accumulated on the anion-exchange membrane (AEM) and within the stack spacers, which required chemical cleaning with HNO_3_ combined with mechanical scrubbing.

### 4.5. Ion Exchange (IE)

Ion exchange represents a purification method particularly suitable for the removal of low concentrations of inorganic salts through the use of ion-exchange resins (Figure 5). In this process, aqueous crude glycerol solutions are passed through a packed bed containing both anion- and cation-exchange resins, enabling the extraction of anions (e.g., Cl^−^) and cations (e.g., Na^+^), while protons and hydroxyl ions are released into the solution, forming water that must subsequently be removed by additional dehydration steps. Raman et al. [19] employed an H^+^-Amberlyst 15 ion-exchange resin to purify industrial crude glycerol previously subjected to an acidification–neutralization pretreatment. The two-step purification process was designed and optimized using the Taguchi method for experimental design. Under optimized conditions, the authors first achieved a glycerol purity of approximately 76.2% through pretreatment, which was then increased to 98.2% after the ion-exchange step by using 40 g of resin, a 60% dilution in methanol, and a flow rate of 15 mL min^−1^. Silva et al. [51] purified a fraction of crude glycerol for subsequent use in the synthesis of glycerol ethers. In their purification procedure, crude glycerol was first acidified using a 4 M H_3_PO_4_ solution and then neutralized with a 6 M NaOH solution. After phase separation, the glycerol-rich fraction was further treated with ion-exchange resins. Specifically, Amberlite IRA 410 (anion-exchange) and Amberlite IRA 120 (cation-exchange) resins were activated with NaOH (4 wt.%) and HCl (5 wt.%) solutions, respectively. Following activation and preliminary filtration, 3 g of IRA 410 and 2 g of IRA 120 were added to a glycerol–water solution (50 vol%) and stirred at room temperature under magnetic agitation. This treatment resulted in a final glycerol purity of approximately 98%. A similar approach was recently adopted by Borówka et al. [52], who investigated the purification of pretreated crude glycerol derived from used cooking oils (UCOs) using five different ion-exchange resins, including three cationic resins (two H^+^-form with gel-type matrix and one H^+^-form with porous matrix) and two anionic resins (both Cl^−^-form with gel-type matrix). The glycerol stream was passed through an ion-exchange bed with a volume of 9 cm^3^ at 60 °C, at flow rates ranging from 2 to 5 cm^3^ min^−1^, corresponding to residence times of approximately 2–5 min. The authors demonstrated that cation-exchange resins were particularly effective in reducing sulfur- and nitrogen-containing compounds in the glycerol mixture. Moreover, superior performance was observed for gel-type resins, whereas porous-matrix resins suffered from pore blockage and progressive deactivation due to fouling phenomena. Based on these observations, it can be concluded that ion-exchange resins are effective in removing salts and impurities from crude glycerol, achieving high purity under optimized conditions. However, issues such as pretreatment requirements, water formation, resin fouling and regeneration costs limit their standalone industrial use. Therefore, ion exchange is most effective as a complementary step within a broader glycerol purification strategy.

### 4.6. Adsorption (AD)

Adsorption is generally applied as the final step in glycerol pretreatment to decolorize and deodorize the purified product using adsorbents with a high specific surface area. Crude glycerol typically has a brownish color (Table 2), usually attributed to the presence of MONG in the mixture. Even after pretreatment, its color may persist as a yellow-transparent shade, depending on the raw material, due to residual low-molecular-weight organic compounds that absorb in the visible range [19]. During adsorption, key parameters to consider include the physicochemical properties of the adsorbent, the adsorbent dosage, the contact time, the temperature and the regeneration of the adsorbent for reuse. The use of commercial activated carbon is very common at this stage of the process due to its relatively low cost and high specific surface area, typically exceeding 650 m^2^ g^−1^ [51,53]. In recent years, however, alternative adsorbents, such as bentonite and activated bentonite [54], as well as bio-based activated carbons derived from renewable sources, including tea waste [55], palm oil biomass [53], and ginger residues [56], have been investigated to further reduce operational costs while promoting a more sustainable circular economy approach. Farid et al. [53] demonstrated that activated carbon derived from palm oil industry biomass is effective in removing up to 89.4% of color-causing molecules from pretreated crude glycerol, performing comparably to commercial activated carbon across different adsorbent dosages (0.5–3 wt.%). In addition, the authors conducted a techno-economic assessment, estimating that approximately 33 m^2^ of this bio-based adsorbent could be produced annually from palm oil residues, potentially reducing costs by around 69% compared to a commercial activated carbon. In a conceptually similar approach, Da Silva et al. [56] investigated adsorbents derived from carbonized ginger residues, also aimed at decolorizing pretreated crude glycerol. By varying parameters such as contact time (60–150 s), temperature (25–45 °C), and crude glycerol concentration in water (30–90 mg/L), and using approximately 50 mg of adsorbent, the authors demonstrated that this material could remove around 75% of the chromophore impurities from the glycerol solution, roughly 20% more than a standard commercial activated carbon.

## 5. Alternative Glycerol Valorization Pathways

**Figure 6:**
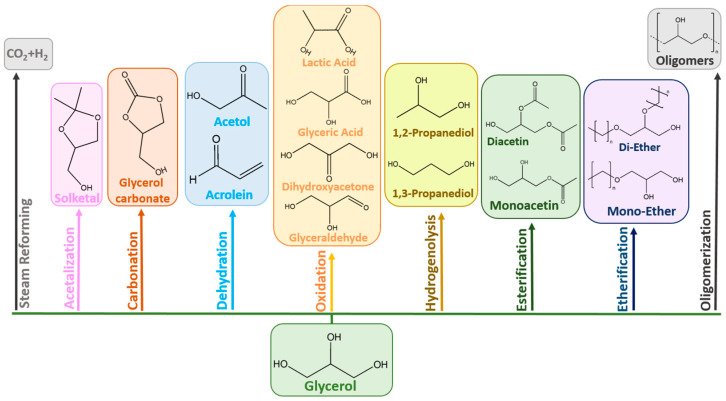
Overview of the possible glycerol valorization pathways.

Once purified to the desired grade (Table 3), glycerol is commercially traded worldwide, as previously discussed, with a global market value of approximately USD 4.8 billion for the final product [25]. A substantial portion of this market (around 25% in 2022) is allocated to the food and beverage sector, where glycerol is employed in more than 1500 direct applications. A similar share is utilized in pharmaceutical and nutraceutical industries, while nearly 40% is consumed in cosmetics and personal care products as a humectant and emollient [25]. Competing, albeit indirectly, with the growing popularity of facial masks and skincare trends, the remaining ~15% of glycerol is directed toward various industrial applications, where the triol serves as a platform molecule for the synthesis of higher value-added chemicals. Among these fine chemicals, acrolein and acetol can be obtained via glycerol dehydration reactions, while monoglyceride esters are produced through esterification of the triol. Solketal, the product of glycerol acetalization with acetone, and glycerol carbonate derived from carboxylation reactions are also noteworthy [57,58]. Considerable attention has been devoted to the selective oxidation of glycerol for the production of glyceraldehyde, glyceric acid, and dihydroxyacetone [59]. Furthermore, etherification reactions leading to mono-, di-, and tri-glycerol ethers represent industrially mature pathways, particularly relevant for the biofuel and cosmetic sectors [60]. Glycerol polymerization has also emerged as a promising route for the synthesis of polymers such as Polyglycerols, Hyperbranched Glycerol (HPG), and glycerol–acrylic polymers [61]. From an energetic perspective, glycerol can undergo steam reforming, acting as a feedstock for syn-gas production and thus as a potential hydrogen carrier molecule [62]. This chapter provides a detailed overview of the most recent advancements reported in the literature regarding the aforementioned processes, as well as others illustrated in Figure 6.

### 5.1. Acetalization

The reaction of glycerol with carbonyl compounds has attracted significant attention due to its potential to produce oxygen-containing molecules that can be employed as surfactants, low-toxicity bio-based solvents, or plasticizers in the polymer industry [57]. Among the various carbonyl-functionalized products that can be formed from glycerol, acetone is by far the most widely used. The acetalization of glycerol with acetone leads to two cyclic products: solketal (4-(hydroxymethyl)-2,2-dimethyl-1,3-dioxolane) and its six-membered ketal isomer (5-hydroxy-2,2-dimethyl-1,3-dioxane), as illustrated in Figure 6, and a molecule of water. The formation of these acetal derivatives occurs through an acid-catalyzed cyclization reaction and can be achieved using homogeneous [63,64] or heterogeneous catalysts. Solid acid catalysts are by far the most studied in the literature, as they generally provide high catalytic activity, good stability, and excellent selectivity, as well as being easier to use in terms of product separation and catalyst recovery [65]. Among the most commonly used solid acids are metal oxides such as WO_x_ and MoO_x_, zeolites, sulfonated silicates [66], and heteropolyacids [67].

**Figure 7:**
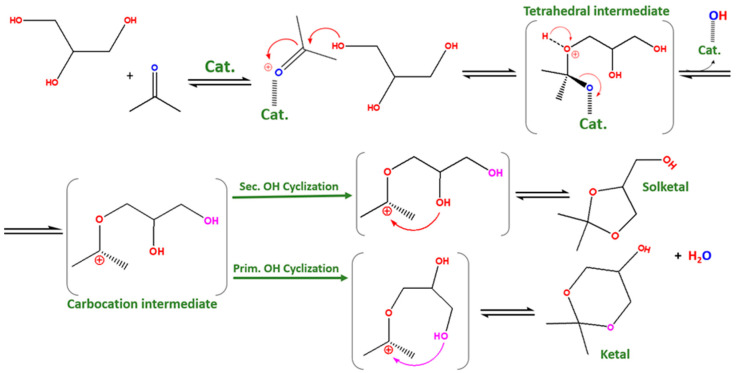
Schematic representation for the formation of solketal and ketal via glycerol acetalization with acetone.

Huang et al. [68] developed a one-pot strategy to synthesize mixed metal oxides (MoO_3_-ZrO_2_) derived from Mo-UiO-66, tuning the molybdenum content (0–30 mol%) to control the dispersion of MoO_3_ species and, consequently, the density of Brønsted and Lewis acid sites. The catalysts were evaluated in the acetalization of glycerol to solketal using 30 mg of catalyst, a glycerol-to-acetone molar ratio of 1:8 (excess acetone being required to shift the equilibrium and suppress solketal hydrolysis), at 50 °C for 10 min. After each run, the catalysts were recovered, calcined at 700 °C for 2 h, and reused for up to five cycles. The best performance was achieved with the 20–MoO_3_-ZrO_2_ catalyst, which reached a glycerol conversion of 89% and a solketal selectivity of 97%. The authors observed no decline in catalytic activity even after the fifth cycle, reporting a solketal yield of around 84%. The most efficient catalyst was also found, via FT-IR pyridine adsorption spectra, to have a particularly high concentration of Lewis acid sites. Through a DFT study, the authors suggest that its high catalytic performance derives from acetone’s propensity to interact more with these groups than with Brønsted acid sites. In fact, the interaction and protonation of acetone is the first step in the glycerol acetalization mechanism (Figure 7). Wang et al. [69] recently modulated Brønsted–Lewis acidity by impregnating spherical alumina nanoparticles with different silica loadings in order to generate new Si-O-Al acid sites. The SA-60 sample, prepared with a silica loading of 60 wt.%, exhibited the highest total acidity (0.4 mmol g^−1^) and consequently delivered the best catalytic performance, achieving approximately 95% glycerol conversion at 60 °C in slightly more than one hour, with a solketal selectivity of 97%. These results clearly highlight the central role of acid site density and strength in governing catalytic activity in glycerol acetalization. Interesting results were also reported by Aguado-Deblas et al. [66] in their study on microwave-assisted solketal synthesis using sulfonic-functionalized silicate catalysts. In this work, the authors investigated the catalytic activity of different amorphous and ordered mesoporous silicates, such as SBA-15 and KIT-6, functionalized with two sulfonic moieties, namely sulfonic acid and propylsulfonic acid groups. The study further compared conventional thermocatalytic synthesis with microwave irradiation, aiming to evaluate potential process intensification effects. The results showed that sulfonic acid introduced at lower loading (5.9 wt.%) provided more effective acid functionalization than propylsulfonic acid, likely due to its stronger intrinsic acidity and lower steric hindrance, which facilitates interaction with surface silanol groups. Among the tested catalysts, SBA-SO_3_H was found to be the most active, achieving about 92% glycerol conversion with 98% solketal selectivity at 80 °C within only 7 min under conventional heating. Under microwave irradiation, the same catalyst delivered comparable performance in just 2 min, using less than 100 W of power and reaching an internal temperature of only 40 °C. Other recent studies have reported similar results using catalytic systems functionalized with sulfonic groups [70,71,72]. For instance, in the study by Sharma et al. [70], five molecules of sulfoisophthalic acid were successfully coordinated to Ru(II) paddlewheel-type metal centers, leading to the formation of metal–organic polyhedra (MOPs) characterized by high surface area and a hierarchical distribution of acid sites. These include Lewis acid sites associated with the metal centers and Brønsted acid sites provided by the sulfonic groups. In the same work, density functional theory (DFT) calculations were employed to elucidate the auxiliary role of the sulfonic groups in the cyclization step leading to solketal formation, thereby rationalizing the high product formation rate (7745 mmol g^−1^ h^−1^). Vannucci et al. [72], on the other hand, reported the sulfonation of commercial zirconia, obtaining a catalyst denoted as Zr-S-400, which achieved approximately 80% glycerol conversion with 86% selectivity. In their study, the authors also investigated the thermodynamic parameters of the reaction by calculating activity coefficients for each species, reporting a standard enthalpy change and Gibbs free energy of reaction of −11.6 kJ·mol^−1^ and 4.0 kJ·mol^−1^, respectively. By applying a pseudo-homogeneous kinetic model and excluding the influence of adsorbed water on the number of active sites, an apparent activation energy of 88.1 kJ·mol^−1^ was determined. Although these catalytic systems exhibit promising performance, microwave-assisted approaches appear to enable comparable or improved results under milder conditions, particularly in terms of reduced reaction times and temperatures compared to conventional thermocatalytic processes. A recent example of this approach is reported by Devasan et al. [71]. In this study, the authors developed a sulfonated catalyst derived from banana peel waste (BP-SO_3_H-15-18-100). Physicochemical characterization confirmed the successful incorporation of sulfonic groups into the catalyst structure. Under microwave-assisted conditions, the catalyst achieved 94.9% glycerol conversion with 97.5% selectivity toward solketal in just 12 min of operative time at 65 °C. Kinetic analysis indicated a non-spontaneous reaction following pseudo-first-order behavior, with an activation energy of 40.23 kJ mol^−1^. The catalyst also exhibited good stability, maintaining 83.48% conversion after five reaction cycles with minimal loss of activity. This behavior suggests that microwave-assisted heating can significantly enhance reaction rates through improved heat transfer and more efficient activation of acid sites for selective solketal production. All the examples discussed are summarized in Table 4.

In conclusion, the reaction of glycerol with carbonyl compounds could be successfully realized by using solid acid catalysts containing a high concentration of Lewis acid sites. Moreover, for achieving high catalytic activity, good stability, and excellent selectivity, a high acetone to glycerol ratio is needed, and glycerol of at least technical purity grade, which would limit the water amount and prevent equilibrium back-shifting. Additional enhancement in the process efficiency for selective solketal production could be achieved by using microwave-assisted heating, which significantly enhances reaction rates through improved heat transfer and more efficient activation of acid sites.

### 5.2. Oxidation

**Figure 8:**
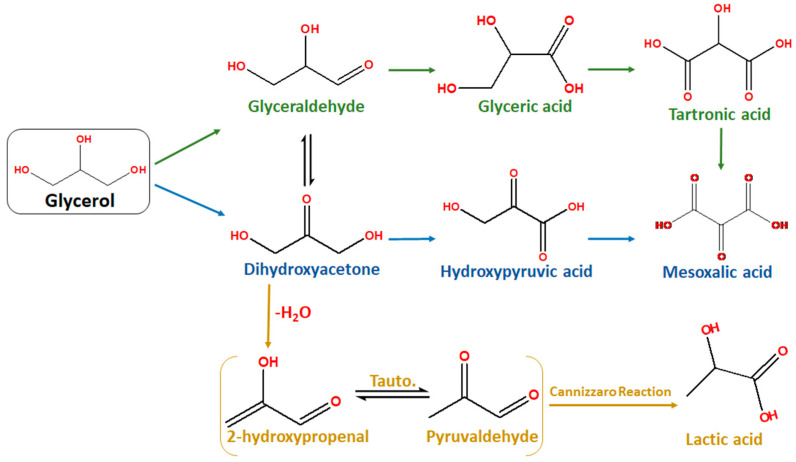
Representation of glycerol oxidative pathways.

The oxidation reaction of glycerol is a multi-stage process that progressively leads to the catalytic oxidation of the hydroxyl groups of the triol molecule, first generating carbonyl functionalizations and culminating in their further conversion to carboxyl groups. When conducted selectively, glycerol oxidation can effectively lead to a wide range of oxygenated products with high added value in a broad spectrum of industrial applications. Dihydroxyacetone and glyceraldehyde are the products obtained by the oxidation of the primary and secondary hydroxyl groups, respectively. The former has applications in the field of cosmetics, particularly in tanning creams, and also acts as a synthetic intermediate for the synthesis of other fine chemicals [59], while the latter has applications in the biochemical and pharmaceutical industries [73]. The carboxylic acids that can be derived from the oxidation and rearrangement of these two molecules are glyceric acid, lactic acid, hydroxyl-pyruvic acid and, in the likely event of C-C bond cleavage reactions, glycolic, oxalic and formic acids, all of which are particularly useful acids in cosmetics and in the chemistry of biodegradable polymers [59]. The oxidation mechanism of glycerol, illustrated in Figure 8, is a multistep process that requires careful modulation of reaction parameters in order to achieve high selectivity toward the desired products.

The presence of acidic or basic species in the reaction medium strongly influences the final product distribution. In particular, under alkaline conditions, glycerol hydroxyl groups can be deprotonated, forming electron-rich alkoxide species that are more reactive and thus more susceptible to oxidation [73]. The presence of bases also promotes the tautomeric equilibrium between glyceraldehyde and dihydroxyacetone through the formation of an enediol intermediate, significantly affecting product selectivity. Conversely, acidic environments suppress hydroxyl deprotonation and consequently hinder tautomerization; however, they often interact with heterogeneous catalysts, potentially limiting their activity or stability [59]. In addition to the reaction medium, glycerol oxidation is highly dependent on the energy source employed to drive the process. Among glycerol valorization reactions, oxidation exhibits the widest range of catalytic approaches, including thermocatalysis, photocatalysis, and electrocatalysis [74]. In thermocatalytic systems, where heat acts as the driving force, relatively high temperatures (typically between 60 and 120 °C) promote the adsorption of glycerol and oxygenated intermediates on heterogeneous catalyst surfaces, which are commonly composed of supported metal or bimetallic nanoparticles. These active phases facilitate the molecular oxygen activation and the C-H and O-H bond cleavage, while the catalyst support, often endowed with acid–base functionalities (Bronsted and/or Lewis), contributes to the stabilization and immobilization of reaction intermediates. The synergistic combination of these surface properties ensures high yields and selectivities toward the desired products. Ke et al. [75] prepared and evaluated a series of bimetallic Au-Pt catalysts supported on MOF-derived transition metal oxides/carbon composites (M_x_O_y_C_z_, where M = Cu, Co, Ce, Mn, Zn, Zr) with the aim of controlling the directional activation of glycerol primary and secondary hydroxyl groups to tune selectivity toward the glyceric acid pathway. In their study, all MOF-derived supports were impregnated with 3 wt.% of an Au-Pt alloy (Au/Pt = 9:1 mol/mol). A total of 52.4 mg of each catalyst was tested in the oxidation of an aqueous glycerol solution (0.1 M, 30 mL total volume) under alkaline conditions (NaOH/glycerol = 2:1 mol/mol) at 60 °C for 2 h in a batch reactor under 1 MPa of oxygen pressure. The best performance was achieved by the Au–Pt/Mn_x_O_y_C_z_ catalyst, which reached complete glycerol conversion with a selectivity of 57.3% toward glyceric acid, the main target product of the study. Surface characterization revealed a pronounced synergistic effect between the Au-Pt alloy and the strong acid–base sites present on the Mn_x_O_y_C_z_ support. This strong metal–support interaction was proposed to promote rapid oxygen activation on the Au-Pt nanoparticles, followed by oxygen radical transfer to the electro-positive Mn^δ+^ surface sites, leading to the formation of reactive peroxide species. These intermediates subsequently interact with glyceraldehyde molecules adsorbed nearby, enabling their fast and selective oxidation to glyceric acid. With a diametrically opposite purpose, Zhang et al. [76] investigated and optimized the morphological engineering of Au nanoparticle-based catalysts supported on mixed copper–zinc oxides (Au/Cu_0.1_Zn_0.9_O), exploring four distinct morphologies: microrod clusters (MRCs), microrods (MRs), microspheres (MSs), and yolk-shell structures (YSs). The aim was to selectively produce dihydroxyacetone under neutral reaction conditions. Catalytic performance was systematically evaluated by varying reaction temperature (60, 80 and 100 °C), oxygen partial pressure (0.5, 1.0 and 1.5 MPa), and reaction time (0.5–3 h). The optimal results were obtained with the Au/Cu_0.1_Zn_0.9_O-MR catalyst at 100 °C, 1 MPa of O_2_, and 2 h of reaction time, achieving complete glycerol conversion with a dihydroxyacetone selectivity of 82.3%. Density functional theory (DFT) calculations further confirmed the critical role of the synergistic interaction between Au nanoparticles and the oxygen-vacancy-rich oxide support in facilitating oxygen dissociation, adsorption, and surface migration of oxygenated intermediates. Among the tested structures, the microrod morphology exhibited the most favorable distribution and accessibility of these active sites, accounting for its superior catalytic performance.

As previously mentioned, glycerol oxidation has recently attracted increasing attention in the electrocatalytic domain. This interest arises from the fact that reactive oxygen-containing radical species, which play a pivotal role in thermocatalytic oxidation pathways, are also naturally generated at the anode of water electrolyzers during the oxygen evolution reaction (OER) under alkaline conditions. This feature allows the oxidation process to proceed without the need for externally supplied pressurized oxygen. Moreover, while water oxidation occurs at a standard equilibrium potential of E^0^ = 1.23 V_RHE_, glycerol electro-oxidation takes place at significantly lower potentials, typically around 0.23 V_RHE_ and 0.32 V_RHE_ for the formation of glyceraldehyde and dihydroxyacetone, respectively [74]. As a result, the glycerol electrooxidation reaction (GEOR) has the potential not only to replace conventional thermocatalytic oxidation routes with reduced energy input, but also to act as an anodic valorization reaction coupled with hydrogen production at the cathode via the hydrogen evolution reaction (HER) [77]. In this configuration, the energetically demanding OER is effectively substituted by the oxidation of an organic substrate, yielding value-added chemicals. As in thermocatalysis, the composition and surface structure of the anodic electrode play a critical role in directing product selectivity. Noble metals such as Pt and Au are among the most commonly employed electrocatalysts due to their high stability and low oxidation overpotentials [77]. However, platinum surfaces, particularly the (110) and (100) crystal facets, are known to promote C-C bond cleavage reactions, thereby reducing selectivity toward C3 products. In contrast, gold predominantly exposes the (111) facet, characterized by low surface energy and high resistance to carbonaceous poisoning owing to its fully occupied d-band [78]. Following this rationalization, Liu et al. [78] developed titanium plate electrodes coated with a sputtered Au layer and further modified by electrodeposition of Pt nanoparticles to selectively produce glyceric acid. The resulting catalyst, denoted as 10Pt/Au/Ti after ten atomic layer electrodeposition (ALED) cycles of Pt, was evaluated for the oxidation of an aqueous glycerol solution (0.1 M) under both acidic (0.1 M HClO_4_) and alkaline (0.1 M KOH) conditions using chronoamperometry at different applied potentials (from 0.9 to 1.5 VRHE). While no oxidation products were detected in acidic media, the alkaline environment enabled high selectivity toward glyceric acid, reaching 93.6% at 0.9 VRHE, together with current densities of approximately 13.6 mA cm^−2^. Nevertheless, despite this high selectivity, glycerol conversion remained limited to 14.6% after two hours of electrolysis. Non-noble metal-based approaches have also been extensively investigated. For instance, Wang et al. [79] developed Cu-doped Ni_3_S_2_ electrocatalysts supported on nickel foam, demonstrating that tuning the copper loading enables both efficient hydrogen evolution reaction (HER) performance (overpotential of 0.37 V at 10 mA cm^−2^) and selective glycerol oxidation toward formic acid. Under alkaline conditions (0.5 M glycerol and 0.1 M KOH) and a constant current density of 100 mA cm^−2^, the system achieved Faradaic efficiencies of up to 85% for formate production, corresponding to approximately 250 mM formate after 10 h of operation. Similarly, Yin et al. [80] employed Mn-doped Ni_7_P_3_ catalysts supported on nickel foam, reaching a Faradaic efficiency of 95.3% for formic acid formation at an applied potential of 1.30 V_RHE_.

Photocatalytic glycerol oxidation has also emerged as a promising and sustainable alternative for glycerol valorization, particularly due to its ability to harness solar energy as the driving force of the reaction. In contrast to thermocatalytic processes, where molecular oxygen and elevated temperatures are typically required, photocatalysis relies on the excitation of a semiconductor material upon light irradiation to generate electron–hole pairs. These photogenerated charge carriers promote the in situ formation of highly reactive oxygen species, such as hydroxyl radicals (•OH) and superoxide anions (which are analogous to those involved in the oxidation pathways previously described. As a result, glycerol oxidation can proceed under mild conditions (ambient temperature and pressure) without the need for externally supplied oxidants, as molecular oxygen dissolved in the reaction medium typically acts as the terminal electron acceptor. From a mechanistic standpoint, the oxidation pathways strongly depend on the interplay between direct hole oxidation and radical-mediated processes. Selective oxidation toward C_3_ products such as dihydroxyacetone or glyceraldehyde is generally favored under controlled conditions that limit radical overproduction, whereas higher irradiation intensities or prolonged reaction times promote C-C bond cleavage, leading to the formation of lower-molecular-weight compounds such as glycolic acid, formic acid, or even CO_2_. Furthermore, the efficiency and selectivity of the process are highly sensitive to catalyst properties, including band gap energy, surface hydroxylation, and the presence of co-catalysts or dopants, which can modulate charge separation and suppress recombination phenomena. In this context, several recent examples have been reported in the literature concerning the selective oxidation of glycerol to formic acid or formaldehyde while suppressing overoxidation to CO_2_. One representative study is that of Fan et al. [81], in which the O_2_•^−^), authors synthesized and characterized an anatase titania photocatalyst surface doped with Co atoms in order to increase the concentration of oxygen vacancies compared to pristine TiO_2_. According to their findings, the introduction of Co species promotes the formation of oxygen vacancies, whose presence was confirmed by EPR analysis, thereby enhancing the adsorption capacity of molecular oxygen and facilitating its activation into superoxide radicals (O_2_•^−^), previously identified as key reactive species in the oxidation process. Furthermore, radical trapping experiments performed using 5,5-dimethyl-1-pyrroline-N-oxide (DMPO) as a scavenger for O_2_•^−^ species provided additional evidence that Co doping improves the separation efficiency of photogenerated charge carriers. The combined effect of enhanced charge separation and increased density of oxygen vacancies leads to more efficient generation of superoxide radicals, which, in synergy with photogenerated holes, promote the cleavage of C-C bonds in glycerol, ultimately yielding C_1_ products such as formic acid. Compared to undoped anatase TiO_2_, the Co-modified catalyst exhibited significantly improved photocatalytic activity, achieving at room temperature nearly complete glycerol conversion (~95%), along with a formic acid selectivity of approximately 57%, while effectively limiting further oxidation to CO_2_. Comprehensive physicochemical characterization indicated that the presence of Co species not only facilitates charge transfer processes but also suppresses electron–hole recombination, thus playing a decisive role in steering the reaction pathway toward selective C-C bond cleavage and the formation of value-added low-molecular-weight products. Surface doping of semiconductor materials, together with the resulting enhancement in visible light absorption and improved charge carrier separation efficiency, represents a key direction toward which research in photocatalytic oxidation is currently evolving. A representative example is the study by Wang et al. [82], in which the authors developed an effective strategy based on the alkalinization and potassium doping of graphitic carbon nitride (C_3_N_4_, denoted as CN) for the selective photocatalytic oxidation of glycerol into value-added chemicals such as dihydroxyacetone (DHA) and glycolic acid (GLA). Density functional theory (DFT) and charge density difference (CDD) analysis, supported by extensive physicochemical characterization, revealed that the introduction of hydroxyl functionalities via the alkalinization treatment significantly alters the charge distribution within the material. In particular, these modifications increase the electron density on the catalyst surface, thereby enhancing its ability to adsorb glycerol compared to both pristine CN and potassium-doped CN without alkalinization. Moreover, the modified catalyst exhibited lower activation barriers for O-H bond dissociation, indicating an increased propensity toward oxidative transformations relative to the unmodified materials. Under ambient conditions, the optimized catalyst, referred to as AKCN, demonstrated a glycerol conversion exceeding 70%, along with a combined selectivity of approximately 70% toward DHA and GLA after 6 h of irradiation. A further development of the concepts discussed above is represented by the work of Kumar et al. [83], who investigated structurally related materials. In this study, the authors designed and systematically evaluated a class of composite nanocatalysts consisting of tellurium nanostructures, specifically nanorods and nanosheets, electrostatically assembled with potassium-doped graphitic carbon nitride (C_3_N_4_), followed by the incorporation of atomically dispersed iridium species. Among the various catalysts tested for photocatalytic glycerol oxidation, the most effective system was identified as the Te-KCN-Ir material (denoted as TeKCNIr), featuring a tellurium content of approximately 10 wt.% and isolated Ir single atoms. Under optimized reaction conditions, this catalyst achieved a glycerol conversion of about 45%, with a remarkable selectivity of 88% toward glyceraldehyde at room temperature under monochromatic visible light irradiation (λ = 450 nm). Given the structural complexity of the material, extensive characterization was carried out to elucidate the origin of its catalytic performance, revealing a strong synergistic interplay among its components. High-resolution electron microscopy and diffraction techniques confirmed the presence of atomically dispersed Ir species anchored onto the K-doped carbon nitride framework, while advanced synchrotron-based spectroscopies indicated that these Ir atoms are stabilized in undercoordinated Ir-N/O environments within the matrix. The incorporation of tellurium, characterized by a relatively narrow band gap, was found to promote efficient charge separation at the heterointerface between Te and the carbon nitride support, thereby enhancing the generation and lifetime of photogenerated charge carriers. In parallel, the isolated Ir sites play a crucial role in directing the reaction pathway, favoring the selective oxidation of glycerol to glyceraldehyde. Mechanistic investigations based on radical scavenging experiments and EPR spectroscopy further indicated that both superoxide (O_2_•^−^) and hydroxyl (•OH) radicals are the primary reactive species involved in the process. Altogether, these findings highlight how the rational integration of heterostructures and single-atom active sites can effectively tune both activity and selectivity in photocatalytic glycerol oxidation systems. These and other examples are detailed in Table 5.

In conclusion, the oxidation of glycerol is a multistep process, highly dependent on the energy source employed, that requires careful modulation of reaction parameters as well as a choice of catalyst composition in order to achieve high selectivity towards a wide range of oxygenated products with high added value. Under alkaline conditions, glycerol hydroxyl groups can be deprotonated, forming more reactive electron-rich alkoxide species, while acidic environments suppress hydroxyl deprotonation and hinder tautomerization. Glycerol oxidation is highly dependent on the energy source employed to drive the process. In thermocatalytic systems, the reaction temperature promotes the adsorption of glycerol on heterogeneous catalyst surfaces where the molecular oxygen activation and the C-H and O-H bond cleavage occur, while the acid–base functionalities of the catalyst support contribute to the stabilization and immobilization of reaction intermediates. In the electrocatalytic approach, the reactive oxygen-containing radical species are naturally generated at the anode of water electrolyzers during the oxygen evolution reaction under alkaline conditions. As a result, the glycerol electrooxidation reaction proceeds without the need for externally supplied pressurized oxygen and can act as an anodic valorization reaction coupled with hydrogen production at the cathode via the hydrogen evolution reaction. Finally, photocatalysis proceeds under mild conditions, as it relies on the excitation of a semiconductor material upon light irradiation to generate electron–hole pairs that promote the in situ formation of highly reactive oxygen species, such as hydroxyl radicals (•OH) and superoxide anions (O_2_•^−^).

### 5.3. Esterification

**Figure 9:**
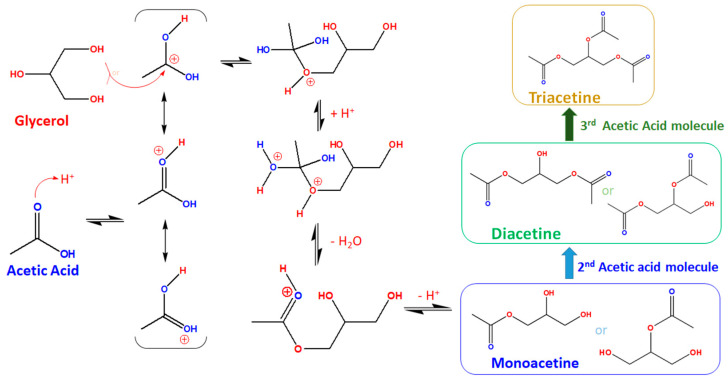
Schematic representation of Fischer’s esterification of glycerol with acetic acid.

The products of glycerol esterification are mono-, di-, and triglycerides, formed through the reaction of the three hydroxyl groups of glycerol with fatty acids or, more commonly, with short-chain carboxylic acids such as acetic acid. In particular, monoglycerides and diglycerides find widespread applications in the pharmaceutical and cosmetic industries as dispersing agents, defoamers, lubricants, and plasticizers [84]. When acetic acid is employed as the esterifying agent, the resulting products are referred to as acetins, namely monoacetin (MAG), diacetin (DAG), and triacetin (TAG). The reaction proceeds through a stepwise acid-catalyzed mechanism, commonly known as Fischer esterification, in which the hydroxyl groups of glycerol are progressively substituted by acetyl functionalities, generating one molecule of water for each ester group formed. As illustrated in the mechanism reported in Figure 9, the acid catalyst initially protonates the carbonyl oxygen of acetic acid, facilitating the nucleophilic attack by one of the hydroxyl groups of glycerol. Following intramolecular rearrangement and elimination of water, the catalyst is regenerated, yielding the monoester MAG. The same sequence subsequently leads to the formation of DAG and ultimately TAG.

However, two major drawbacks inherently limit the selectivity and techno-economic feasibility of this process. First, glycerol contains two primary hydroxyl groups and one secondary hydroxyl group, all capable of acting as nucleophiles. As a result, the formation of positional isomers is unavoidable, leading to typically observed MAG isomer ratios close to 2:1, with analogous behavior for DAG formation [84]. This multiplicity of isomers complicates product purification and restricts selectivity toward specific mono- or diesters. Moreover, although glycerol acetylation proceeds through a consecutive reaction pathway, experimental evidence suggests that the final esterification step toward triacetin is kinetically hindered. Steric effects and reduced accessibility of the remaining hydroxyl group in partially esterified intermediates often limit the third nucleophilic substitution, leading to the accumulation of mono- and diacetins rather than full conversion to TAG. A second major limitation arises from the equilibrium nature of Fischer esterification. To drive the reaction toward higher ester yields, elevated temperatures, excess acetic acid, and continuous removal of the produced water are generally required. These operational constraints significantly increase energy demand and process costs, thereby limiting large-scale implementation. Various acidic catalysts have been employed for glycerol esterification. Historically, homogeneous mineral acids such as HCl and H_2_SO_4_ have been widely used due to their high catalytic activity [85]; nevertheless, their corrosive nature, hazardous handling, and the generation of large quantities of inorganic salt wastes during neutralization and product recovery render them environmentally unfriendly and economically unattractive, with limited potential for catalyst reuse. Significant efforts have been devoted to replacing conventional mineral acids in glycerol esterification with more environmentally friendly acidic catalysts offering higher thermal stability and improved reusability. Among these alternatives, Keggin-type heteropolyacids (HPAs), such as H_3_PW_12_O_40_, H_4_SiW_12_O_40_, and H_3_PMo_12_O_40_, have been extensively investigated. These compounds exhibit well-defined metal–oxygen polyhedral structures characterized by strong Brønsted acidity arising from proton delocalization over the polyanionic framework. In addition to their high thermal stability, their acidic properties can be finely tuned through metal substitution or partial proton exchange, making them attractive catalysts for esterification reactions [86]. In this context, Da Silva et al. [87] compared silicotungstic and phosphotungstic acids with their partially exchanged Zn(II) and Sn(II) counterparts in the acetylation of glycerol. Reactions were carried out at 60 °C for 8 h using a glycerol-to-acetic acid molar ratio of 1:3 and a catalyst loading of 0.1 mol%. The authors observed that Sn-based catalysts exhibited higher activity than Zn-based ones. Among them, the phosphotungstic-derived catalyst Sn_3/2_PW_12_O_40_ achieved a glycerol conversion of approximately 65% with a pronounced selectivity toward diacetin (74%). This enhanced performance was attributed to the higher density of active acidic sites resulting from the synergistic interaction between tin cations and the phosphotungstic acid (TPA) framework. A similar modulation of acidic properties in TPA was investigated by Ertaş et al. [88], who synthesized metal-exchanged derivatives containing Fe, Cr, Cu, and Ni cations. Their study revealed a progressive decrease in Brønsted acidity accompanied by an increase in Lewis acidity, following the order TPA > Fe-TPA > Cr-TPA > Cu-TPA > Ni-TPA, as confirmed by pyridine-FTIR analysis. The metal-exchanged catalyst with the highest retained Brønsted acidity achieved a glycerol conversion of 96% with high selectivity toward diacetin (68%), while triacetin formation remained limited to approximately 10%. Another research direction currently under investigation involves coupling the strong acidity of heteropolyacids (HPAs) with heterogeneous high-surface-area inert supports or solid acids with mixed acidic properties, aiming to achieve simultaneously high conversions, controlled selectivity, and easy catalyst separation and reuse. For instance, Yadav et al. [89] prepared a series of solid acid catalysts by combining a cesium-substituted TPA with an acidic clay support, namely montmorillonite K-10, through incipient wetness impregnation. The most active catalyst, containing approximately 20 wt.% of Cs_2.5_H_0.5_W_12_O_40_ supported on K-10 (denoted as 20%Cs-DTP-K10), achieved a glycerol conversion of 92.5% after 4 h at 120 °C using a glycerol-to-acetic acid molar ratio of 1:9 and an agitation speed of 800 rpm. Under these optimized conditions, however, product selectivity was distributed between monoacetin (46%) and triacetin (48%). The authors further examined the influence of reaction temperature (110–140 °C), stirring rate (600–1000 rpm), and reactant molar ratio (1:3, 1:6 and 1:9), demonstrating that the 20%Cs-DTP-K10 catalyst maintained nearly constant activity over three consecutive reaction cycles, indicating good stability and reusability. In an effort to enhance selectivity toward triacetin formation, Baingam et al. [90] synthesized a series of propylsulfonic acid-functionalized KIT-6 mesoporous silica materials (PA-KIT-6) and compared their catalytic performance with conventional solid acids of known acidity, namely protonated zeolite H-ZSM-5 (surface acid site density = 0.95 μmol H^+^ m^−2^) and the ion-exchange resin Amberlyst-15 (88 μmol H^+^ m^−2^). The most effective catalyst, P-20PA-KIT-6, obtained through post-synthetic grafting of mercaptopropyltrimethoxysilane (MPTMS) followed by oxidation of thiol groups into sulfonic acid functionalities, exhibited a high surface area of 544 m^2^ g^−1^ and a surface acid site density of 0.46 μmol H^+^ m^−2^. In glycerol acetylation performed at 115 °C with a glycerol-to-acetic acid molar ratio of 1:9 and a catalyst loading of 2.83 wt.%, complete glycerol conversion was achieved along with a triacetin selectivity of 52%. Notably, this performance surpassed that of H-ZSM-5 (23.7%) and Amberlyst-15 (33.6%), despite their higher nominal acidity. The authors attributed this improvement to the superior accessibility and dispersion of active acid sites within the mesoporous framework of KIT-6. This interpretation was further supported by comparing P-20PA-KIT-6 with its directly synthesized analog (D-20PA-KIT-6), which, although possessing a higher acid site density, displayed inferior catalytic activity due to poorer textural properties, lower surface area, and reduced pore volume, ultimately limiting molecular diffusion and reactant accessibility. From the perspective of enhancing active site accessibility to improve catalytic selectivity, particularly toward diacetin (DAG) formation, Bravo-Sanabria et al. [91] developed and compared several modified MOF-808 catalysts, in which the introduction of surfactants during synthesis altered the mesoporous structure of the framework and facilitated molecular diffusion during the sequential acetylation steps. Specifically, MOF-808 was synthesized in the presence of three different surfactants: cetyltrimethylammonium bromide (CTAB), sodium dodecyl sulfate (SDS), and Pluronic P123 (PLU), combined with propionic acid as a modulator. Among the obtained materials, the P123-modified sample (MOF-808-PLU) exhibited an 8% increase in total surface area compared to conventionally synthesized MOF-808, reaching 1384 m^2^ g^−1^, along with a significant enhancement of the mesoporous fraction, which accounted for approximately 23% of the total surface area (around 431 m^2^ g^−1^). This improved mesoporosity translated into a remarkably high selectivity toward DAG formation, reaching 84%. Additional studies have explored the use of metal oxides, mixed metal oxides, and acid-functionalized high-surface-area carbon supports as heterogeneous catalysts for glycerol acetylation. For instance, Doukeh et al. [85] synthesized and evaluated SnFe_2_O_4_ nanocatalysts, while Termezi et al. [92] investigated various Fe and Ni loadings supported on activated carbon. Abida et al. [93] prepared Ce(IV)-based catalysts supported on sulfated TiZrO_4_ (TiZrO_4_@SO_4_^2−^), achieving high DAG selectivity (summarized in Table 6). Similarly, Farisya et al. [94] reported promising results using TiO_2_ catalysts with different acidity levels induced via sulfonation (Table 6). Collectively, these studies highlight the critical role of catalyst textural properties and acid site accessibility in steering glycerol acetylation toward desired partial esterification products.

It can be concluded that the esterification of glycerol requires the use of a high-surface-area catalyst that allows high dispersion and density of strong acid sites. Collectively, these studies highlight the critical role of catalyst textural properties and acid site accessibility in steering glycerol acetylation toward desired partial esterification products.

### 5.4. Etherification

**Figure 10:**
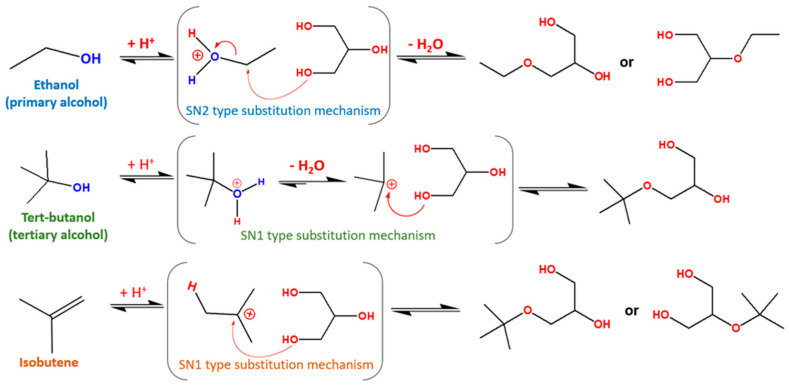
Schematic representation of three possible mechanisms of acid-catalyzed etherification of glycerol.

Glycerol etherification involves the acid- or base-catalyzed condensation of glycerol hydroxyl groups with alcohols or alkenes through dehydration pathways, leading to the formation of branched oxygen-containing molecules with extended carbon chains. These glycerol-derived ethers have found applications as pharmaceutical intermediates, lignocellulosic solubilization promoters, non-ionic surfactants [96], and fuel blending agents aimed at improving diesel combustion performance or increasing the octane number in the gasoil (especially the tri-substituted glycolic ethers) [97]. When alcohols are employed as etherifying agents, the reaction generally proceeds through a multistep acid-catalyzed dehydration condensation mechanism (schematically illustrated in Figure 10), in which the hydroxyl groups of glycerol are sequentially substituted by alkyl groups. This stepwise process leads to the formation of mono-, di-, and tri-alkyl glycerol ethers, commonly referred to as MGE, DGE, and TGE, respectively. Due to the presence of two primary hydroxyl groups and one secondary hydroxyl group in the glycerol molecule, etherification can occur at different positions, resulting in the formation of structural isomers for both MGE and DGE. Glycerol etherification can be catalyzed under either acidic or basic conditions; however, acid catalysis is generally preferred due to its faster kinetics and higher conversion levels [51]. Although strong mineral acids can effectively promote the reaction, their use often leads to undesired side reactions. Excessively strong acidic environments favor not only the self-etherification of the alcohol reactant, forming symmetrical ethers, but also glycerol self-condensation, resulting in polyglycerol oligomers. To mitigate byproduct formation and facilitate catalyst recovery, solid acid catalysts are, therefore, commonly employed. Nevertheless, despite significant improvements achieved with heterogeneous catalysts, controlling selectivity toward specific ether products (MGE, DGE, or TGE) remains challenging. Moreover, as glycerol etherification is intrinsically a dehydration equilibrium reaction, the continuous formation of water negatively affects both reaction equilibrium and catalyst stability. Efficient water removal and the development of hydrothermally stable catalysts are thus essential requirements for process intensification and industrial implementation [97]. Short-chain alcohols such as ethanol, isopropanol, tert-butanol, and related low-molecular-weight alcohols are most commonly employed as etherifying agents in glycerol etherification processes. Reactions are typically conducted at temperatures ranging from 90 to 120 °C, while the glycerol-to-alcohol molar ratio plays a crucial role in determining both conversion levels and product selectivity toward mono-, di-, and tri-substituted glycerol ethers. Silva et al. [51], for instance, purified a crude glycerol sample and subsequently subjected it to etherification with ethanol and isopropanol using varying glycerol-to-alcohol molar ratios (1:3, 1:6, and 1:12). Amberlyst-15 resin (2 g) was employed as a solid acid catalyst at 110 °C. For both alcohols, the highest glycerol conversion was achieved at a molar ratio of 1:12, reaching 97.5% with ethanol and 85.2% with isopropanol. A marked difference in product distribution was also observed depending on the alcohol structure. Owing to the higher reactivity and lower steric hindrance of the primary alcohol, reactions conducted with ethanol exhibited significant selectivity toward di- and tri-ether products (approximately 37% and 43%, respectively). In contrast, etherification with the secondary alcohol isopropanol resulted in a strong preference for mono-glycerol ethers, with selectivities exceeding 95%, while only negligible amounts of di-ethers and no tri-ethers were detected. In order to enhance catalyst hydrophobicity and facilitate the rapid removal of water formed during the reaction, thereby shifting the equilibrium toward ether formation, Ausavasukhi et al. [98] developed cerium-modified Beta zeolites and compared their performance with conventional acidic zeolites for the synthesis of tert-butyl glycerol ethers. The incorporation of cerium species into the H-Beta zeolite framework, together with charge compensation at ion-exchange sites, was found to increase the hydrophobic character of the material in proximity to Brønsted acid sites, thereby enhancing its catalytic performance. Etherification experiments were conducted using a glycerol-to-tert-butanol molar ratio of 1:4 at a relatively mild temperature of 90 °C for approximately 2 h. Among conventional zeolites, H-Beta exhibited superior catalytic activity compared to other acidic zeolites, following the order H-Beta > H-Mordenite > H-Y > H-ZSM-5. H-Beta achieved a glycerol conversion of 67% with a selectivity toward mono-tert-butyl glycerol ether (MTBG) of 92%, which was primarily attributed to its higher concentration of Brønsted acid sites (approximately 1.20 mmol g^−1^) and larger pore structure, which enhances the accessibility of the acid sites (Table 7). The Ce-modified H-Beta catalyst (CeHBeta) further outperformed the parent zeolite despite possessing a slightly lower Brønsted acidity (1.09 mmol g^−1^), reaching a glycerol conversion of 81%, with selectivities of 86.5% toward MTBG and 13.5% toward di-ethers. No formation of tri-ethers was detected, which was ascribed to the reduced reactivity of tert-butanol as a tertiary alcohol and to the substantial steric hindrance associated with tert-butyl substituents. The catalyst also retained its catalytic performance over at least five consecutive reaction cycles. The strategy of increasing the hydrophobic character of solid acid catalysts to enhance etherification efficiency was further explored by Ausavasukhi et al. [99], who modified carbon-based solid acids derived from palm kernel shells through different hydrothermal sulfonation treatments with sulfuric acid. Among the prepared materials, the catalyst denoted as PKS-160 exhibited the highest glycerol conversion and a selectivity toward MGE of approximately 83%, outperforming the other sulfonated carbon samples. Physicochemical characterization revealed that the hydrothermal treatment applied to PKS-160 (1 g of palm kernel shells treated with 20 mL of H_2_SO_4_ at 160 °C for 24 h in an autoclave) resulted in a higher density of acid sites, as well as enhanced surface hydrophobicity compared to the parent materials. According to the authors, the increased hydrophobicity not only promoted etherification activity by facilitating water desorption from the catalytic surface but also accounted for the improved catalyst stability, which was maintained over at least four reaction cycles. Da Silva et al. [60] investigated the effect of Sn^2+^ incorporation into three different Keggin-type heteropolyacids, namely H_3_PW_12_O_40_, H_3_PMo_12_O_40_, and H_4_SiW_12_O_40_, aiming to elucidate how increasing tin loadings influence acid properties and catalytic performance in glycerol tert-butyl etherification. Among the three systems, the tin-doped phosphomolybdic acid-based catalyst exhibited the most pronounced improvement upon Sn incorporation, displaying a nearly linear increase in glycerol conversion with increasing Sn loading. In particular, the Sn_1.5_PMo_12_O_40_ catalyst achieved approximately 70% glycerol conversion at 100 °C with a glycerol-to-tert-butanol molar ratio of 1:4, while maintaining over 65% selectivity toward MGE after four hours of reaction. Nevertheless, a selectivity of around 15% toward glycerol oligomers was also observed, although significantly lower than that detected for the other catalysts in the series. Acid site characterization revealed that this catalyst possessed a higher concentration of medium-strength Brønsted and Lewis acid sites, which were identified as key contributors to the adsorption–activation–desorption steps governing the etherification mechanism.

Glycerol direct etherification with alcohols via dehydration represents an efficient valorization pathway, with most heterogeneous acid catalysts enabling glycerol conversions above 80% under relatively mild conditions, particularly in the presence of excess alcohol. Nevertheless, product distributions are consistently dominated by mono- and di-alkyl glycerol ethers, while the formation of tri-substituted ethers (TEG) remains scarce or negligible across most systems. This behavior reflects intrinsic steric and kinetic limitations associated with the progressive substitution of the glycerol backbone, which increasingly hinders access to the remaining hydroxyl groups. Catalyst design strategies based on enhanced mesoporosity optimized acid site distribution, and increased surface hydrophobicity have successfully improved activity and selectivity toward MEG and DEG but have not yet overcome the fundamental barriers to high TEG yields. Additionally, the condensation nature of the reaction leads to continuous water formation, shifting reaction equilibria and potentially deactivating acid sites, thus requiring excess alcohol feed or hydrophobic catalysts to sustain performance. In the literature, an alternative acid-catalyzed etherification route has been developed to achieve high yields and selectivities toward higher glycerol ethers, particularly di- and tri-glycerol ethers. Although this approach entails higher technological complexity and operational costs, it represents a chemically elegant and mechanistically advantageous strategy. This process relies on the etherification of glycerol via tertiary carbocations generated from isobutene (or other olefins). As schematically illustrated in Figure 9, a Brønsted acid catalyst—most commonly a sulfonated ion-exchange resin—protonates the C=C bond of isobutene, leading to the formation of a highly stable and reactive tertiary carbocation. Subsequently, one of the hydroxyl oxygen atoms of glycerol nucleophilically attacks the tert-butyl carbocation through an SN1-type mechanism, resulting in the formation of mono-tert-butyl glycerol ether. The advantages of this pathway are evident. First, no water is formed as a byproduct, effectively shifting the reaction equilibrium toward ether formation and avoiding catalyst deactivation associated with water accumulation. Second, despite the steric hindrance imposed by the tert-butyl group, the reaction exhibits faster kinetics compared to alcohol-based etherification routes, owing to the absence of intramolecular rearrangements and the involvement of a highly reactive carbocationic intermediate. An additional and industrially relevant feature of this system is that, under acidic liquid-phase conditions, isobutene and tert-butanol coexist in a proton-transfer equilibrium. This enables a co-etherification strategy employing both isobutene and tert-butanol simultaneously, thereby mitigating key drawbacks of isobutene-based etherification, namely catalyst fouling and olefin dimerization.

In this context, Liu et al. [97] investigated the etherification of glycerol to tert-butyl glycerol ethers using a commercial ion-exchange resin (NKC-9, ion-exchange capacity 4.7 mol H^+^ kg^−1^). The authors systematically evaluated the effects of temperature (70–100 °C) and, most importantly, of different glycerol:isobutene:tert-butanol molar ratios. Their results demonstrated not only the high efficiency of isobutene-mediated etherification, achieving up to ~80% combined yield of di- and tri-glycerol ethers at 90 °C, but also the effectiveness of the co-etherification approach, which, although slightly reducing the overall yield of highly substituted ethers (~55%, Table 8), almost completely suppressed isobutene dimerization. With respect to solid acid catalysts, largely analogous to those employed in conventional glycerol etherification, a comprehensive benchmark study was reported by Bozkurt et al. [100]. In this work, more than 70 acidic catalysts were collected, systematically characterized, and evaluated under identical reaction conditions for glycerol etherification with tert-butanol (selected examples are summarized in Table 8). The investigated catalyst set encompassed a wide range of solid acids, including ion-exchange resins such as Amberlyst-15 (4.50 mmol H^+^ g^−1^) and Amberlyst-36 (5.00 mmol H^+^ g^−1^), zeolites Y (0.14 mmol H^+^ g^−1^), Beta (0.22 mmol H^+^ g^−1^), and ZSM-5 (SiO_2_/Al_2_O_3_ = 23; 0.58 mmol H^+^ g^−1^), as well as heteropolyacids such as silicotungstic acid (TSA, 1.95 mmol H^+^ g^−1^) and phosphotungstic acid (TPA, 4.04 mmol H^+^ g^−1^). However, despite the breadth of the catalyst library investigated, the study also underscores a critical limitation: high acid site density alone does not guarantee high selectivity toward di- and tri-glycerol ethers, as diffusional constraints and steric effects increasingly dominate at higher degrees of substitution. In addition to these catalytic considerations, the techno-economic assessment reported by López-Suárez et al. [101] highlights a further major limitation of isobutene-mediated glycerol etherification, namely the intrinsic cost of the reagent itself, together with the increased technological complexity of the reaction system. In particular, the process necessarily operates at elevated pressures to maintain isobutene in the liquid phase, resulting in higher capital and operational expenditures. The authors demonstrated that operating at the high isobutene concentrations required to enhance selectivity toward DGE and TGE leads to reagent costs exceeding the current market value of the resulting glycerol ethers, thereby undermining the economic competitiveness of the process.

In conclusion, it can be summarized that the following requirements ought to be fulfilled in order to successfully use the etherification valorization route: presence of a hydrothermally stable and hydrophobic solid acid catalyst containing strong acid sites together with an alcohol or alkene with high reactivity and low steric hindrance, while preserving a relatively low glycerol to alcohol/alkene ratio.

### 5.5. Dehydration

**Figure 11:**
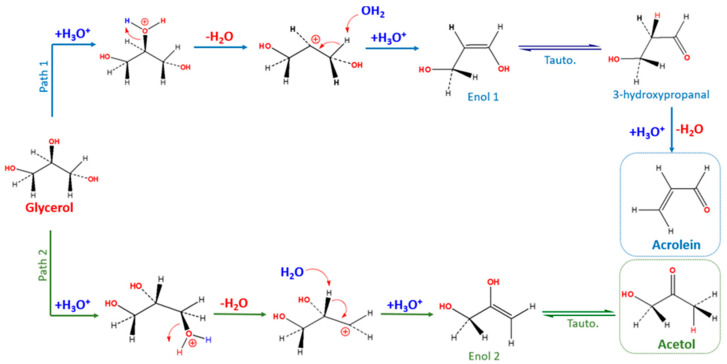
Reaction pathways occurring during glycerol dehydration.

The acid-catalyzed dehydration of glycerol can be formally classified as an elimination reaction, involving the removal of either a secondary hydroxyl group to form acrolein or the primary hydroxyl group to form acetol. However, unlike classical E1 or E2 eliminations, glycerol dehydration proceeds through a complex multistep pathway comprising hydroxyl protonation, water elimination, the formation of carbocation-like intermediates, and subsequent rearrangements (shown in Figure 11). These steps are strongly governed by the nature, strength, and distribution of the catalyst’s acidic sites, as well as by the operating conditions, which collectively determine the reaction selectivity and final product distribution. Among the products accessible through glycerol dehydration, acrolein (2-propenal) is the most industrially relevant. Despite being a toxic, flammable, and highly irritating compound, acrolein is a key precursor for the production of acrylic acid and acrylate polymers [102]. It is employed in the pharmaceutical industry for the synthesis of methionine and imidacloprid [103] and is also used as a biocide and aquatic herbicide. Alternatively, dehydration through the primary glycerol’s hydroxyl groups yields acetol (1-hydroxy-2-propanone), which, although often regarded as an intermediate, can be further dehydrated to acrolein [102] or valorized as a platform molecule. Acetol finds applications as an intermediate in cosmetic formulations, including tanning solutions [59], and as a precursor for the production of 1,2- and 1,3-propanediols, widely used as antifreeze components and plasticizers.

Translating these mechanistic insights into selective catalytic systems remains challenging, and recent experimental studies have focused on engineering acidic catalysts capable of balancing activity, selectivity, and stability under dehydration conditions. Solid acid catalysts have been extensively investigated for glycerol dehydration, with recent studies mainly focusing on phosphates, heteropolyacids, modified zeolites, and metal oxides. Among these, phosphate-based catalysts have attracted growing attention due to their tunable Brønsted acidity and comparatively improved hydrothermal stability. Chen et al. [103] developed a series of iron phosphate (FePO_4_) catalysts calcined at different temperatures, using thermal treatment as a descriptor to modulate the relative Brønsted acid strength. The goal was not only to achieve high glycerol conversion and acrolein selectivity but also to mitigate one of the major drawbacks of solid acids in glycerol dehydration, namely, catalyst deactivation under hydrothermal conditions. Catalytic tests were carried out in a continuous-flow fixed-bed stainless-steel tubular reactor at temperatures between 280 and 340 °C, using 800 mg of catalyst and a feed rate of 4.2 g h^−1^ of a 10 wt.% aqueous glycerol solution. The FePO_4_ catalyst calcined at 600 °C (HT-600) exhibited complete glycerol conversion at 300 °C after 2 h on stream, with an acrolein selectivity of 93.2% and a carbon balance of 98.7%. However, prolonged operation led to progressive catalyst deactivation. After 6 h on stream, glycerol conversion decreased by approximately 5.3%, accompanied by a drop in acrolein selectivity to about 80%. Structural characterization revealed that catalyst deactivation was primarily associated with the transformation of iron phosphate into iron pyrophosphate (Fe_2_P_2_O_7_). Nevertheless, the authors demonstrated that a post-reaction calcination step could largely restore the catalytic activity, indicating that deactivation was at least partially reversible. In a related study, Su et al. [102] investigated boron phosphate (BPO_4_) catalysts, systematically tuning the concentration of B^3+^- and P^4+^-related defects through variation of the calcination temperature, with the aim of modulating Brønsted acid site density and strength. Among the samples tested, the catalyst calcined at 1000 °C (BPO_4_-1000) showed the best performance, achieving nearly complete glycerol conversion at 320 °C, with acrolein and acetol selectivities of approximately 80% and 14%, respectively, and a carbon balance close to 98%. Minor formation of acetaldehyde (~1.7%) was also detected, decreasing to ~0.8% as the catalyst’s calcination temperature increased from 400 to 1000 °C. Notably, BPO_4_-1000 displayed exceptional long-term stability, maintaining full glycerol conversion over 425 h on stream, with only a moderate decline in acrolein selectivity to ~75% and a carbon balance of ~95%. This outstanding stability was attributed to several factors, including the enhanced resistance of boron species to aqueous leaching after high-temperature calcination, despite the strongly hydrothermal reaction environment. Density functional theory (DFT) calculations further suggested that, under operating conditions, in situ Brønsted acid sites are preferentially formed that are chemo-selective toward the acrolein-forming pathway while exhibiting relatively weak adsorption of acrolein itself, thereby reducing the propensity for coke formation and catalyst deactivation. Condotta et al. [104] performed a systematic comparative study between two major classes of solid acid catalysts, namely zeolites and metal oxides, for the gas-phase dehydration of glycerol to acrolein. The study systematically investigated and optimized key operating parameters, including reaction temperature (320–450 °C), glycerol concentration in water (20–80 wt.%), liquid hourly space velocity (LHSV: 0.37, 0.57, 1.12, and 1.50 h^−1^), and corresponding feed flow rates (0.18, 0.36, 0.54, and 0.72 mL min^−1^). Catalytic tests were carried out in the gas phase using a fixed-bed Haber–Bosch-type reactor capable of accommodating approximately 10 g of catalyst. Six different solid acids were evaluated, namely Al_2_O_3_, fluorinated alumina (F-Al_2_O_3_), MgO/Al_2_O_3_, Nb_2_O_5_, the zeolite H-ZSM-5, and the zeolite US-HY. Selected results obtained under optimized conditions for maximum acrolein yield are reported in Table 9. The study showed that, under the investigated conditions, glycerol conversion was consistently close to 100% for all catalysts. However, increasing reaction temperature promoted the formation of undesired byproducts, thereby limiting the achievable acrolein yield. Among the catalysts tested, metal oxides exhibited the best overall performance, with Nb_2_O_5_ reaching an acrolein yield of 78% and F-Al_2_O_3_ achieving up to 80%. In contrast, the zeolitic catalysts H-ZSM-5 and US-HY provided lower acrolein yields, approximately 62.2% and 64.6%, respectively. Nevertheless, US-HY reached comparable yields at significantly shorter residence times than H-ZSM-5, suggesting a higher intrinsic activity, likely associated with its larger pore size and enhanced molecular diffusion. The study highlights that, beyond achieving full glycerol conversion, the key challenge in glycerol dehydration lies in balancing acid strength, residence time, molecular pore diffusion and thermal severity to maximize acrolein yield while minimizing secondary reactions.

In line with the critical role of molecular diffusion within the catalyst framework, alongside the distribution and density of acidic active sites in maximizing acrolein yields and determining catalyst stability, Yu et al. [105] conducted a detailed study on the combined impact of porosity and acidity on the deactivation behavior of ZSM-5 zeolites in glycerol dehydration. To decouple the individual contributions of acidity and porosity, the authors first investigated three conventional microporous ZSM-5 zeolites with different SiO_2_/Al_2_O_3_ ratios (SAR = 25, 70, and 500, denoted as Z25, Z70, and Z500, respectively). These materials exhibited strictly microporous structures and Brønsted acid site densities inversely proportional to their silica content. From a catalytic standpoint, glycerol conversion was found to increase with increasing SAR, i.e., with decreasing acid site density. The high-silica Z500 sample achieved up to 84% glycerol conversion during the initial hours of reaction, with an acrolein selectivity of approximately 79%. Nevertheless, all three catalysts experienced a sharp and rapid deactivation after approximately two hours on stream, with glycerol conversion over Z500 dropping to nearly 40%. Post-reaction characterization of the spent catalysts revealed severe pore blockage caused by carbonaceous deposits, identified as polyglycerols and polycyclic aromatic hydrocarbons. These species originated from the limited diffusional transport of reactants and intermediates within the narrow microporous channels of ZSM-5, ultimately leading to the rapid poisoning of Brønsted acid sites and catalyst deactivation. To mitigate this issue, the authors introduced hierarchical mesoporosity into ZSM-5 via controlled alkaline treatment and subsequent realumination. The resulting catalysts exhibited enlarged pore diameters (>10 nm) and a more homogeneous distribution of acidic sites. These structural modifications significantly improved catalytic performance, enabling the modified samples AT(0.005)-Z500 and AT-Z70 to sustain approximately 97% glycerol conversion for at least 10 h on stream, with negligible activity loss and acrolein selectivities ranging between 70% and 75%.

In conclusion, glycerol dehydration process requires the use of a solid acid catalyst with high stability to acid sites leaching under hydrothermal conditions, readily accessible acid sites and porosity that allows fast transportation of reactants and products in order to minimize secondary reactions and limit catalyst deactivation due to carbonaceous deposits.

### 5.6. Oligomerization

**Figure 12:**
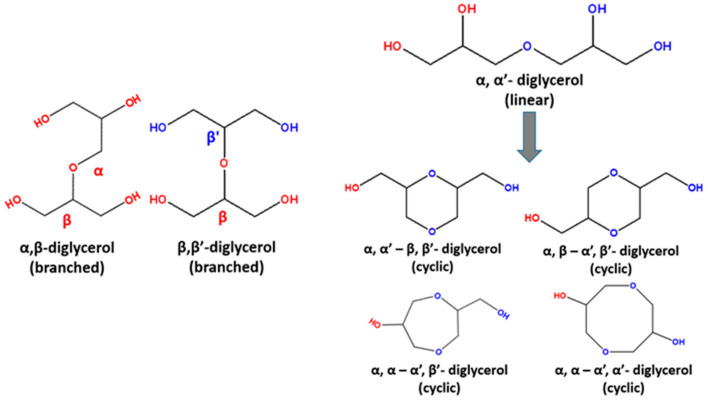
Possible glycolic dimers formed during the glycerol oligomerization process.

In a manner closely analogous to the etherification reactions discussed previously, glycerol, owing to its triol functionality, can undergo self-condensation with another glycerol molecule to form ether-linked dimeric species commonly referred to as diglycerols. Depending on whether the initial condensation occurs at one of the two primary hydroxyl groups or at the secondary hydroxyl group, the resulting diglycerol may exhibit either a linear or a branched structure (depicted in Figure 12). Diglycerol still contains four free hydroxyl groups and can therefore further react with additional glycerol molecules, leading to the formation of higher oligomers. As a consequence, a complex mixture of linear, branched, and cyclic oligoglycerols can be generated, with the final product distribution being strongly dependent on the reaction conditions and the nature of the catalyst employed [106]. This structural tunability broadens the potential application of oligoglycerols in several fields, including polymer additives, surfactants, lubricants, and cosmetic formulations. From a catalytic standpoint, glycerol oligomerization can be promoted under both acidic and basic conditions. However, due to the competitive dehydration pathway leading to acrolein formation under acidic catalysis, this reaction is more commonly investigated using basic catalysts. In addition, reactions are often carried out under inert atmosphere in order to suppress oxidative side reactions, especially at the elevated temperatures typically employed (240–260 °C). Regardless of whether acidic or basic catalysis is used, glycerol oligomerization is formally an etherification reaction and therefore produces water as a byproduct. The accumulation of water shifts the reaction equilibrium, and it must be controlled or continuously removed to sustain oligomer formation. Moreover, as the degree of oligomerization and cross-linking increases, the viscosity of the reaction medium rises significantly, which may lead to non-uniform temperature profiles and diffusion limitations toward the active sites of the catalyst, so in this reaction, stirring is highly recommended [107].

From a catalytic standpoint, glycerol oligomerization under basic conditions has been investigated using both homogeneous catalysts, such as Na_2_CO_3_, K_2_CO_3_, or ionic liquids, and heterogeneous systems including alkali-doped metal oxides, hydrotalcites and other layered double hydroxides (LDH), and also fluoroperovskites [108]. Among recent homogeneous approaches, Kansy et al. [106] reported the synthesis and characterization of oligoglycerols containing more than four repeating glycerol units by systematically varying the concentration of Na_2_CO_3_ as a catalyst. Their work specifically addressed one of the main challenges of this reaction, namely the regioselective production of defined oligoglycerol fractions and a controlled degree of glycerol polymerization. Experiments were conducted at 230 °C for 12 h under reduced pressure (0.4 bar), using catalyst loadings between 0.67 and 3.33 wt.% relative to 400 mL of deaerated glycerol under magnetic stirring. Under all tested conditions, glycerol conversion exceeded 90%, with a clear preference toward higher-molecular-weight oligomers (>350 g mol^−1^), corresponding to chains containing more than four glycerol units. Detailed characterization of the isolated oligomeric fractions revealed the formation of two atypical terminal functionalities: one class bearing an additional terminal hydroxyl group, and another containing a carboxylic acid group at the chain end. The latter likely originates from secondary oxidation or rearrangement processes occurring under the harsh reaction conditions. In a subsequent study, Kansy et al. [109] also explored ionic liquids as catalysts for the selective production of low-molecular-weight oligoglycerols. Two main classes were investigated: 3-alkyl-1-methylimidazolium bromides with varying alkyl chain lengths (ethyl, dodecyl, tetradecyl) and 1-dodecyl-N,N,N-triethylammonium acetate. The ammonium-based ionic liquid exhibited the best performance, achieving glycerol conversions of about 94% at 180 °C within 3 h, with pronounced selectivity toward diglycerol and triglycerol. Although heterogeneous basic catalysis offers clear advantages in terms of catalyst recovery and process sustainability, it is often associated with significant limitations. These include rapid catalyst deactivation after the first use, alkali leaching promoted by high operating temperatures, dissolution of the active phase, and loss of crystallinity, particularly in zeolitic materials. Such drawbacks are further exacerbated when low-purity glycerol feedstocks are employed. To address some of these limitations, Barros et al. [110] prepared Mn-Al layered double hydroxides (LDHs) via rehydration of mixed oxides and evaluated both calcined samples and materials obtained through different rehydration procedures in glycerol oligomerization. The most effective Mg-Al LDH catalyst (denoted MAU05P) was obtained through a sequential rehydration treatment of the mixed oxides, first in water and subsequently in acetic acid. Under optimized conditions (240 °C, 8 h reaction time), this catalyst achieved approximately 64% glycerol conversion in the first reaction cycle, with about 35% selectivity toward diglycerols and roughly 55% toward higher-molecular-weight oligomers. The catalyst also demonstrated good stability after the first cycle, provided that periodic rehydration treatments assisted by ultrasonication were applied to reconstruct the lamellar LDH structure and restore accessible basic active sites. In a subsequent study, Barros et al. [107] further investigated cobalt-modified LDHs by comparing Co(acac)_2_-impregnated Mg–Al LDH with Co-Al mixed oxides and Mg-Al mixed oxides (MgAl MO). Cobalt impregnation significantly improved catalytic performance: the Co/MgAl LDH achieved glycerol conversion of approximately 68%, with around 67% selectivity toward oligoglycerols. However, unlike the MAU05P sample, the Co/MgAl LDH exhibited rapid deactivation upon recycling, with conversion dropping to about 26% in the second cycle. This loss of activity was attributed to blockage of active basic sites by strongly adsorbed oligoglycerol species formed during the reaction. High selectivity toward diglycerol (up to 92%) was reported by Khumho et al. [111] using Mg-Al layered double oxides (Mg_1_Al_1_/LDO/CaCO_3_) derived from natural dolomite through hydrothermal treatments. The physicochemical modifications introduced during catalyst preparation effectively tuned the balance between acidic and basic sites, which helped to suppress excessive glycerol polymerization and favor dimer formation. However, this improved selectivity came at the expense of catalytic activity: glycerol conversion decreased from about 77% for the untreated dolomite to approximately 52% for the modified catalyst.

It can be concluded that the glycerol oligomerization process is difficult to control, characterized by relatively moderate conversion levels due to equilibrium limitations, increased viscosity of the reaction medium, and progressive diffusion constraints. Hence, its practical implementation is strongly constrained. However, some promising results could be obtained when using heterogeneous catalysts containing balanced acid–base sites that are stable to leaching, favor dimer formation, and are prone to recovery procedures. All the discussed results are summarized in Table 10.

### 5.7. Carbonation

**Figure 13:**
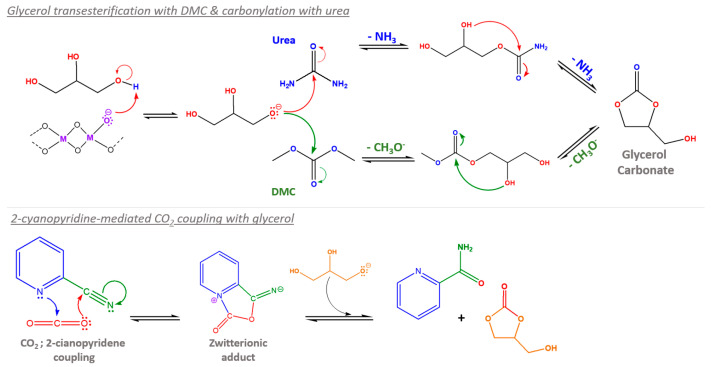
Schematic representation of possible synthetic strategies used to synthesize glycerol carbonate.

The carbonation of glycerol to produce glycerol carbonate (GC) represents one of the most valuable valorization pathways for this highly versatile platform molecule. Glycerol carbonate has attracted considerable attention due to its broad range of applications, including its use as a plasticizer in cementitious mixtures [112], as a precursor for binders in lithium metal batteries (LMBs) [113], as a building block together with itaconic acid for the production of photopolymers used in 3D printing [114], as a low-environmental-impact plasticizer for polylactic acid (PLA) [115], and as a green solvent. Several carbonate sources can be employed for GC synthesis, and the reaction mechanism varies depending on the carbonylating agent used. When glycerol reacts under basic catalysis with organic carbonates such as dimethyl carbonate (DMC), diethyl carbonate (DEC), ethylene carbonate (EC), or propylene carbonate (PC), the reaction proceeds through a transesterification mechanism (Figure 13). This pathway yields one molecule of glycerol carbonate along with two equivalents of alcohol released from the original carbonate reagent. In contrast, when urea is used as the carbonylating agent, the reaction is more accurately described as a carbonylation process, proceeding through the elimination of two molecules of ammonia (Figure 13). More recently, increasing attention has been directed toward routes based on the direct coupling of glycerol with CO_2_, or with CO/O_2_ gas mixtures. The CO_2_-based pathway is particularly attractive from a sustainability perspective, as it enables the simultaneous utilization of two waste-derived feedstocks: glycerol from biodiesel production and carbon dioxide, a major greenhouse gas. Furthermore, this approach has the potential to significantly reduce the costs associated with the use of organic carbonates as carbonating agents [116].

Transesterification between glycerol and organic carbonates is generally carried out in the presence of basic catalysts and has been extensively investigated using both homogeneous and heterogeneous catalytic systems. Among the various carbonylating agents, dimethyl carbonate (DMC) represents a particularly attractive reagent, as it is widely regarded as a green chemical due to its low toxicity, biodegradability, and environmentally benign synthesis routes. From a homogeneous catalysis perspective, this reaction can be promoted by a variety of inorganic and organic bases, including NaOH, KOH, and triethylamine. For instance, Pan et al. [117] investigated the use of NaOH under different reaction conditions and reported optimized performance at 70 °C for 2 h with a DMC-to-glycerol molar ratio of 4. Under these conditions, glycerol conversion reached 77%, while the selectivity toward glycerol carbonate (GC) remained relatively low at only 23%. Such modest selectivity highlights a well-known limitation of strongly basic homogeneous catalysts, which tend to promote side reactions, particularly the decomposition of GC into glycidol. In addition to selectivity issues, homogeneous catalytic systems suffer from intrinsic drawbacks, including difficulties in catalyst separation, limited recyclability, and increased operational costs, all of which represent significant barriers for industrial scale-up. To overcome these limitations, considerable efforts have been devoted to developing heterogeneous catalytic alternatives. Recent studies have explored a wide range of solid catalysts, including single and mixed metal oxides, hydrotalcites, and zeolites (selected examples are summarized in Table 11). For example, Gómez-Garduño et al. [58] synthesized a series of Li_2−x_Na_x_ZrO_3_ solid solutions (0 < x < 2) and evaluated their catalytic performance. The most active composition, Li_1.8_Na_0.2_ZrO_3_, achieved approximately 98% glycerol conversion and a GC yield of 97.3% at 80 °C after 3 h using a relatively low DMC-to-glycerol molar ratio of 1.5. The catalyst also demonstrated stability over three reaction cycles. However, a sharp decrease in yield to about 48% was observed during the fourth cycle, and XRD analysis revealed an almost complete loss of crystallinity after the fifth run, indicating significant structural instability under reaction conditions. These findings suggest that, despite the excellent initial activity, long-term durability remains a critical issue for such materials. Kowalska-Kuś et al. [118] optimized GC synthesis from biodiesel-derived crude glycerol using DMC as the carbonylating agent and hierarchical ZSM-5 zeolites, while also investigating the effect of ultrasonic cavitation. The study compared two Si/Al ratios (27 and 55, denoted SN27 and SN55) and their subsequent alkaline-treated counterparts obtained via NaOH or KOH dealumination. The alkaline treatments increased the mesoporosity of the materials, reduced their relative acidity, and enhanced the density of basic sites compared to the parent zeolites. The most effective catalyst, KOH-treated SN55, achieved approximately 98% glycerol conversion, with a GC yield of 95% at 90 °C after 4 h with a DMC/glycerol molar ratio of 5 (using commercial glycerol). Notably, similar catalytic performances were obtained at a lower temperature (70 °C) when an ultrasonic probe was applied, highlighting the beneficial role of cavitation in improving mass transfer and catalytic efficiency. Under the same reaction conditions, when crude glycerol was used as the feedstock, the catalyst achieved only about 55% glycerol conversion. This decrease in catalytic performance was attributed to the poisoning of active sites and pore blockage caused by MONG (matter organic non-glycerol) impurities present in the crude feed. Another example of product selectivity variation as a function of catalyst synthesis conditions was reported by Argüello et al. [119]. In this study, the authors prepared quaternary Cu-Ni-Mg-Al mixed metal oxides derived from Mg-Al layered double hydroxides (LDHs) and calcined them at different temperatures to investigate how thermal treatment influences catalytic performance in the transesterification reaction. Among the examined calcination temperatures (450–750 °C), the sample treated at the lowest temperature, MMO-Cu_15_Ni_15_-T_450_, exhibited the highest surface area (249 m^2^ g^−1^), the highest concentration of strong basic sites (total basicity: 3.33 mmol g^−1^), and the lowest density of acidic sites among the series (10.29 μmol g^−1^). According to the authors, this optimized acid–base balance explains why this catalyst achieved the highest selectivity and yield toward glycerol carbonate (95.3% and 84.6%, respectively) while maintaining a relatively low selectivity toward glycidol (~4.7%).

Although recent results on the transesterification of glycerol with dimethyl carbonate (DMC) appear highly promising in terms of selectivity and product yield, important process limitations remain. Alkyl carbonates are synthetically produced reagents, and their use in excess is often necessary to shift the equilibrium toward product formation. Combined with the energy-intensive separation and recycling steps, this may significantly undermine process economics at a large scale [120]. In recent years, an alternative pathway has gained increasing attention, namely the use of urea as the carbonylating agent. Compared to DMC, urea is less expensive and, in principle, releases two molecules of ammonia during the reaction, which could be captured and subsequently reacted with CO_2_ to regenerate urea. Although this route is thermodynamically less favorable than transesterification and therefore requires higher operating temperatures, it remains more viable than the direct coupling of glycerol with CO_2_ (discussed later). Notably, the reaction typically achieves high glycerol conversions and good glycerol carbonate yields. As in the DMC route, both homogeneous and heterogeneous catalysts have been investigated, with most studies focusing on the balance and role of acid–base surface sites in determining conversion and product distribution. Several representative examples are reported in Table 11. Among heterogeneous systems, zinc-based catalysts have attracted particular interest due to their high catalytic activity and excellent glycerol carbonate yields. A representative example is the ZnO@Co-MOF core-shell catalyst reported by Wang et al. [120]. In this work, the Co/Zn molar ratio was varied to tune the ZnO shell thickness and consequently modulate the surface acid–base properties. The most active catalyst (Z@C-0.2) exhibited the highest relative concentration of acidic sites together with moderate basicity, which enabled a glycerol carbonate yield of approximately 92.6% using a particularly advantageous urea/glycerol molar ratio of 1. Thakkar et al. [121] prepared zinc phosphonate catalysts via precipitation using different molar ratios of zinc and sodium alendronate (Na-ALN/Zn = 0.5, 1, and 2). Under optimized reaction conditions (150 °C, 10 wt.% catalyst loading, and a urea-to-glycerol molar ratio of 1:1), the most active catalyst (ALN1Zn2) achieved approximately 86% glycerol conversion after 7 h of reaction, with a glycerol carbonate yield of 83%. The catalyst also exhibited good stability, showing only about a 9% decrease in conversion after four consecutive reaction cycles. In the same study, the authors proposed a reaction mechanism supported by in situ FTIR experiments. Analysis of the vibrational bands observed on the catalyst surface indicated the formation of a carbamate intermediate, which was identified as a key species in glycerol carbonate formation. This finding contrasts with previous reports suggesting an alternative pathway involving a metal-isocyanate intermediate.

While the urea-based route has demonstrated good efficiencies for glycerol carbonate production, it still requires relatively high reaction temperatures and management of the ammonia generated during the process. For this reason, growing attention has been directed toward alternative carbonation strategies, particularly the direct coupling of glycerol with CO_2_, which is considered especially attractive from both sustainability and waste-valorization perspectives. The rationale behind the presumed sustainability of this process lies in its potential to simultaneously valorize two waste streams: greenhouse CO_2_ and surplus glycerol from biodiesel production, converting them into a high-value fine chemical such as glycerol carbonate. However, the reaction is thermodynamically constrained, with a positive Gibbs free energy change (ΔG° ≈ +23.9 kJ mol^−1^), and the equilibrium is strongly limited by water formation. Moreover, because the process operates under high CO_2_ pressures, water cannot be readily removed by evaporation, like in other glycerol upgrading reactions discussed previously. Instead, a dehydrating agent must be added to chemically scavenge water and shift the equilibrium toward product formation. In practice, nitriles such as acetonitrile or cyanopyridines are commonly employed for this purpose [116]. According to the study by Sarkar et al. [122] on Cu/In_2_O_3_/ZnO catalysts, cyanopyridines are more effective than acetonitrile because they play a dual role: they act both as dehydrating agents and as promoters for CO_2_ activation. Under identical reaction conditions, three cyanopyridine isomers were evaluated, and 2-cyanopyridine provided the highest glycerol carbonate yield (24.4%). Similarly, Ke et al. [123] screened several metal oxides (e.g., CuO, NiO, Co_3_O_4_, ZrO_2_, Al_2_O_3_) for the direct coupling of CO_2_ with glycerol, again using 2-cyanopyridine as the dehydrating agent. Among the tested catalysts, CuO exhibited the best performance, achieving 89% glycerol conversion and 69.4% selectivity toward glycerol carbonate at 120 °C, 3 MPa CO_2_ pressure, and 5 h reaction time. This relatively high activity, compared to most recent literature reports, may arise from synergistic effects. In CuO, copper is predominantly present as stable Cu^2+^ species associated with variable oxygen lattice vacancies, which likely provide Lewis acidic sites capable of adsorbing both glycerol and the lone pair of 2-cyanopyridine. Beyond simply facilitating CO_2_ activation, the latter is plausibly involved in forming a metastable zwitterionic adduct generated by CO_2_ insertion between the pyridinic nitrogen and the nitrile group of 2-cyanopyridine (as illustrated in Figure 13). The formation of this intermediate is believed to significantly lower the energy barrier for glycerol carbonate formation.

**Table 11:** Recent breakthroughs in the synthesis of glycerol carbonate using various synthetic strategies.

Catalyst	Reaction Conditions	Glycerol Conv. (%)	GC Selectivity (%)	Reusability (Cycles)	Ref.
Glycerol transesterification with organic carbonates					
Li_1.8_Na_0.2_ZrO_3_	– Temperature: 80 °C – Time: 3 h – DMC/GL molar ratio: 1.5 – 10 mol% of catalyst	98.2	99.1	3	[58] 2026
SN(55)_KOH	– Temperature: 70 °C – Time: 5 min. – DMC/GL molar ratio: 5 – 10 wt.% of catalyst – Ultrasonic probe used	98	97	4	[118] 2026
3CaO/TiO_2_	– Temperature: 90 °C – Time: 3 h – DMC/GL molar ratio: 4 – 3 wt.% of catalyst	99.3	96.4	6	[124] 2021
MMO-Cu_15_Ni_15_-T_450_	– Temperature: 85 °C – Time: 4.5 h – DMC/GL molar ratio: 2 – 7.5 wt.% of catalyst	88.8	95.3	-	[119] 2025
Glycerol carbonylation with urea					
In_0.66_TPA	– Temperature: 140 °C – Time: 4 h – Urea/GL molar ratio: 2.3 – 10 wt.% of catalyst	69.4	98.8	4	[125] 2023
Z@C-0.2	– Temperature: 140 °C – Time: 3 h – Urea/GL molar ratio: 1 – 4 wt.% of catalyst	92.6	100	5	[120] 2026
ALN1Zn2	– Temperature: 150 °C – Time: 7 h – Urea/GL molar ratio: 1 – 10 wt.% of catalyst	86.3	96.1	4	[121] 2026
Glycerol coupling with CO_2_					
2%Cu/In_2_O_3_ /ZnO	– Temperature: 150 °C – Time: 5 h – CO_2_ pressure: 5 MPa – 1.5 wt.% of catalyst – 2-cyanopyridine/Glycerol: 3	38	64.4	5	[122] 2025
0.3ZnO-CaO-SBA-15	– Temperature: 110 °C – Time: 4 h – CO_2_ pressure: 3 MPa – 6 wt.% of catalyst – acetonitrile/Glycerol: 3	64	76.2	5	[126] 2026
CuO	– Temperature: 120 °C – Time: 5 h – CO_2_ pressure: 3 MPa – 3 wt.% of catalyst – 2-cyanopyridine/Glycerol: 3	89	69.4	7	[123] 2024

In conclusion, carbonation of glycerol to produce glycerol carbonate can be realized by using various carbonate sources. The transesterification of glycerol with organic carbonates requires the use of solid basic catalysts that are structurally stable under the reaction conditions and contain predominantly strong basic sites and well-developed textural properties. The use of urea as a carbonylating agent usually results in high glycerol conversions and good glycerol carbonate yields; however, this approach requires the use of higher operating temperatures, as it is thermodynamically less favorable than transesterification. Finally, the direct coupling of glycerol with CO_2_ seems the most promising route due to its potential to simultaneously valorize two waste products; nevertheless, its thermodynamic constraint and the water formation require the use of an additional dehydrating agent. In this case, the use of a catalyst that synergistically reacts with the dehydrating agent could significantly decrease the energy barrier and produce relatively high activities for glycerol carbonate formation.

### 5.8. Hydrogenolysis

**Figure 14:**
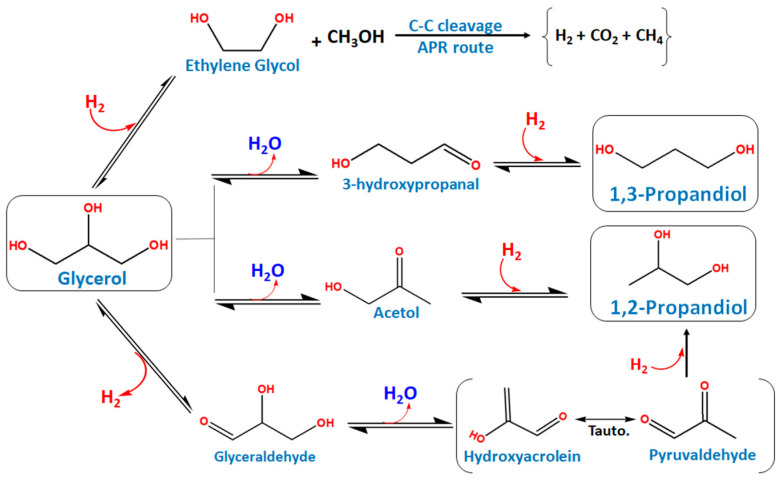
Schematic representation of the possible pathways taken by glycerol during the hydrogenolysis reaction.

The hydrogenolysis of glycerol can be conceptually regarded as a selective pathway within the broader network of reactions occurring during the catalytic processing of aqueous glycerol solutions under subcritical conditions, commonly referred to as aqueous phase reforming (APR). In aqueous-phase systems, glycerol conversion generally proceeds through a common set of elementary steps, including dehydrogenation to carbonyl intermediates, dehydration, hydrogenation, C-C bond cleavage, and redox transformations on bifunctional catalytic sites. As a result, APR, oxidation, and hydrogenolysis should not be viewed as entirely distinct processes but rather as different outcomes within the same interconnected reaction network, whose selectivity is mainly governed by catalyst properties and the prevailing reaction environment. In classical APR, the reaction pathway is dominated by extensive C-C bond cleavage and reforming steps, leading predominantly to the formation of hydrogen and carbon dioxide. In contrast, aqueous-phase hydrogenolysis (APH) selectively suppresses C-C scission while promoting C-O bond cleavage and hydrogenation sequences. A key factor governing the transition between these regimes is the hydrogen balance within the reaction environment: hydrogen may be externally supplied or generated in situ through glycerol dehydrogenation and reforming reactions, and its availability strongly dictates whether the system evolves toward deep reforming or selective hydrogenolysis pathways (Figure 14). Under conditions favoring hydrogenolysis, glycerol conversion is steered toward the formation of value-added C_3_ products, primarily 1,2-propanediol and, under suitable catalytic environments, 1,3-propanediol [127]. These products have a wide range of industrial applications, making the APH process particularly attractive for glycerol valorization. In addition, the use of aqueous reaction media allows less stringent requirements on feed purity, enabling the direct processing of semi-refined glycerol streams. Specifically, 1,2-propanediol is currently regarded as a practical substitute for ethylene glycol in the formulation of biodegradable antifreeze fluids, de-icing agents, coolants, and heat-transfer fluids [128]. Owing to its low toxicity, this compound also finds applications in the cosmetic, food, and pharmaceutical industries, as well as serving as a humectant in tobacco products [129]. In contrast, although significantly less toxic than ethylene glycol, 1,3-propanediol is less commonly used for food applications. However, as a linear diol, it is an important building block for the synthesis of polymers such as polyurethanes (PUs) and polytrimethylene terephthalate (PTT) [130], and is also used as an additive in solvents, adhesives, laminates, and resins [131].

As illustrated in Figure 14, glycerol hydrogenolysis in the presence of hydrogen gas may proceed through multiple competing cleavage pathways. These include C-C bond scission routes leading to the formation of ethylene glycol and methanol, followed by subsequent reforming reactions that ultimately generate gaseous products such as H_2_, CO_2_, and CH_4_. Alternatively, if glycerol undergoes an initial selective dehydroxylation step, different intermediates can be formed depending on the position of the hydroxyl group involved. Dehydroxylation at one of the primary alcohol groups leads to the formation of acetol, whereas removal of the secondary hydroxyl group produces 3-hydroxypropionaldehyde. Subsequent hydrogenation of the carbonyl functionality in these intermediates yields 1,2-propanediol and 1,3-propanediol, respectively. As can be readily understood, for reasons analogous to those observed in other glycerol transformations such as esterification and etherification, the intrinsic structural asymmetry of glycerol plays a decisive role. Since two primary hydroxyl groups are present compared to only one secondary hydroxyl group, the formation of 1,2-propanediol (1,2-PD) is both kinetically and thermodynamically favored during aqueous-phase hydrogenolysis (APH). This preference is particularly pronounced under hydrogen-rich conditions, elevated temperatures, and when using catalysts featuring strongly Brønsted-acidic supports combined with metals exhibiting high hydrogen spillover capability. In addition to these pathways, a fourth plausible mechanism may operate under mildly oxidative surface environments or specific catalytic conditions. In this route, partial oxidation of a primary hydroxyl group leads to glyceraldehyde formation, followed by dehydration and hydroxyacrolein–pyruvaldehyde tautomerization. Subsequent reduction steps ultimately yield 1,2-propanediol (Figure 14). For the reasons outlined above, the selective synthesis of 1,3-propanediol represents the most significant challenge in glycerol hydrogenolysis. Nevertheless, achieving high selectivity toward 1,2-propanediol is also not trivial, as several competing reactions can reduce yields. These include direct C-C cleavage pathways producing ethylene glycol, over-hydrogenolysis leading to shorter-chain alcohols, and base-catalyzed side reactions such as oxidation or Cannizzaro-type processes that can generate lactic or propionic acid derivatives. Therefore, precise modulation of operating parameters, including temperature, acid–base properties of catalyst supports, and the nature and density of active metallic sites, is essential to achieve optimal selectivity and yields toward either propanediol isomer. For the selective production of 1,2-propanediol (1,2-PD) via aqueous-phase hydrogenolysis (APH), the choice of the active metal is particularly critical. Catalysts based on metals with high efficiency for C-O bond hydrogenation–dehydrogenation sequences (such as Cu, Zn, Sn, and to some extent Ni) are generally preferred over noble or rare metals, which tend instead to promote undesired C-C bond cleavage. A recent example is the study by Ge et al. [132], which investigated CuFeZnAl-based hydrotalcite-derived catalysts (HDTs) with different M^2+^/M^3+^ cation ratios in the mixed oxides after reductive pretreatment. Batch reactor experiments demonstrated that the Cu/Zn molar ratio plays a key role in ensuring a fine dispersion of Cu^0^ nanoparticles. Moreover, controlling the Fe/Cu ratio revealed that intermediate iron contents (around 18 wt.%) generate a synergistic interaction between Fe_2_O_3_ and CuO, lowering the reduction activation energy of copper oxide and thus facilitating its reduction. The study also included an extensive screening of reaction parameters, such as temperature, reaction time, catalyst loading, glycerol concentration in aqueous solution, hydrogen pressure, and recyclability. Under optimized conditions, the Cu_35_Fe_18_Zn_45_Al_2_-HDT catalyst exhibited the best performance, achieving nearly complete glycerol conversion (~100% at 30 wt.%) with a 1,2-PD selectivity of 96.4% after 6 h at 230 °C. However, the catalyst showed limited recyclability (about three cycles), associated with a significant decrease in specific surface area and growth of Cu particle size after reaction. This deactivation was mainly attributed to copper sintering and ZnO agglomeration under high-temperature conditions. Increasing the aluminum content in the CuFeZnAl-HDT system was shown to improve stability by strengthening metal–support interactions, thereby helping preserve dispersion and structural integrity during reuse. Beyond intrinsic composition, the preparation method also plays a crucial role in determining catalytic performance. For instance, Mane et al. [128] demonstrated that the calcination temperature, as well as the reduction temperature used to prepare CuAl_2_O_4_ catalysts, strongly affects metal dispersion, acid–base site distribution, and textural properties, which in turn directly influence catalytic outcomes. In this study, equimolar Cu-Al mixed metal oxides were prepared at different calcination temperatures ranging from 300 to 1000 °C. The catalyst treated at the highest temperature (CuAl-1000) was found to be the least active, mainly due to the complete removal of Brønsted acid sites caused by the severe thermal treatment, as well as to extensive sintering of Cu^0^ crystallites, which significantly reduced the catalytically active metallic surface area. By contrast, catalysts calcined at lower temperatures retained both Brønsted acidity and smaller Cu^0^ and CuAl_2_O_4_ crystallite sizes. The most performant sample, calcined at 400 °C (CuAl-400 (DC)), exhibited the best catalytic behavior, achieving about 60% glycerol conversion with a 1,2-PD selectivity close to 94%. The examples discussed above mainly refer to systems operating with an exogenous hydrogen supply. However, recent literature also reports promising advances based on catalysts capable of generating sufficient hydrogen in situ through parallel glycerol APR pathways coupled with the water–gas shift reaction (CO + H_2_O → CO_2_ + H_2_), thereby enabling a self-sustained APH process. A representative example is the work by Reynoso et al. [133], who investigated nickel aluminate catalysts doped with vanadium or boron to increase the density of oxophilic Lewis acid sites. After a prior reduction treatment, the resulting materials were evaluated under continuous-flow conditions using a fixed-bed reactor. The catalytic screening was carried out using three different doping levels of V and B on nickel aluminate (0.5, 1, and 3 wt.%). Both vanadium and boron modifications led to substantial improvements in catalytic performance compared to the pristine NiAl catalyst, mainly due to the formation of more effective surface redox pairs. In both series, however, the highest doping level (3 wt.%) resulted in poorer performance, which was attributed to the formation of large, poorly dispersed crystallites with limited accessibility and reduced exposure of acid–base active sites to the reacting molecules. The catalysts 1B-NiAl and 1V-NiAl showed stabilized glycerol conversions of about 84% and 80%, respectively, and maintained stable activity for at least 28 h on stream, whereas the undoped NiAl catalyst experienced a marked deactivation, with conversion dropping to about 35% over the same period. In terms of product yields, these catalysts produced 1,2-PD at 26% and 33.6%, respectively, with hydroxyacetone, ethylene glycol, and ethanol identified as the main byproducts. The relatively modest 1,2-PD yields observed in the absence of externally supplied hydrogen can be rationalized by the fact that autogenously generated H_2_ necessarily originates from parallel reforming reactions catalyzed by metallic sites (in this case Ni^0^). Under such conditions, the formation of C-C cleavage products such as ethylene glycol and light alcohols becomes largely unavoidable. An additional limitation arises from hydrogen-consuming gas-phase reactions, including CO_2_ methanation and hydrogenation to light alkanes, which further decrease the effective hydrogen availability in the reaction environment and consequently suppress 1,2-PD yields. For these reasons, the performances reported in this study, as well as in other recent autogenous-hydrogen APH systems [127,134], summarized in Table 12, tend to exhibit a common limitation: while catalyst stability and glycerol conversion can be relatively high, the selectivity and yield toward 1,2-PD remain intrinsically constrained by the unavoidable competition between reforming, hydrogen generation, and hydrogen consumption pathways.

The selective synthesis of the 1,3-propanediol (1,3-PD) isomer via aqueous-phase hydrogenolysis (APH) is considerably more challenging than that of 1,2-PD. As previously discussed, hydrogenolysis of the secondary hydroxyl group in glycerol is intrinsically hindered by both steric constraints and the higher intrinsic reactivity of the primary hydroxyl groups [135]. To overcome these limitations and enhance 1,3-PD yields, reaction conditions typically require more moderate temperatures than those employed for 1,2-PD production, while catalysts must exhibit strong metal–support interactions and supports rich in oxygen vacancies [136]. Among the most intensively investigated systems are catalysts containing Pt as the active metallic site combined with W-based cocatalysts, either incorporated into the support or present as interacting secondary species. For instance, Li et al. [135] examined the influence of WO_x_-SiO_2_ interactions on 1,3-PD selectivity. They demonstrated that in Pt/W-SiO_2-x_ catalysts, weaker WO_x_-SiO_2_ interactions lead to higher Pt-WO_3_ coordination numbers, which promote a stronger hydrogen spillover effect. This, in turn, enhances the formation of H-WO_3_ species that facilitate glycerol activation. The Pt/W-SiO_2_-700 catalyst, characterized by the weakest WO_x_-SiO_2_ interaction, achieved about 70% glycerol conversion with a 1,3-PD yield of 43.5% at a relatively mild temperature of 140 °C under optimized conditions (Table 12). Further advances in Pt/WO_3_-based systems were recently reported by Jiang et al. [136], who investigated the role of crystal-plane engineering in prism-shaped TiO_2_ nanorods used as supports for Pt/WO_3_ catalysts. Their study revealed a clear “crystal plane-defect-active site” relationship: rutile TiO_2_ {110} planes, which are rich in oxygen vacancies and Ti^3+^ sites, strongly promote the anchoring and dispersion of Pt and WO_x_ nanocrystallites. The catalyst with the highest {110}/{111} exposure ratio (Pt-WO_x_/RTNR-453) benefited from improved metal dispersion, enhanced hydrogen spillover, and in situ generation of Brønsted acid sites by WO_x_. Under optimized conditions at 150 °C, it achieved 96.7% glycerol conversion and a 1,3-PD yield of 58.6% after 24 h of reaction. The main secondary product was 1-propanol (34.8% selectivity). Despite these promising results, most of the recent literature on selective 1,3-PD synthesis still revolves around incremental modifications of Pt/WO_x_-type systems (some examples in Table 12). This reflects a fundamental constraint: the key factors governing selectivity toward 1,3-PD, namely low reaction temperatures and high hydrogen pressures, require catalysts capable of very efficient H_2_ chemisorption and spillover under mild conditions. In practice, only noble metals such as Pt, Ir, and Re [137,138] currently provide sufficient hydrogen activation to sustain the hydrogenolysis pathway under these conditions.

In conclusion, hydrogenolysis of glycerol promotes C-O bond cleavage and hydrogenation sequences towards the formation of 1,2-propanediol and 1,3-propanediol, while hydrogen is either externally supplied or generated in situ through glycerol dehydrogenation and reforming reactions, thus enabling a self-sustained process; however, it is at the expense of lower yields due to the unavoidable competition between reforming, hydrogen generation, and hydrogen consumption pathways. The selective production of 1,2-propanediol requires precise modulation of operating parameters, including temperature, acid–base properties of catalyst supports, and the nature and density of active metallic sites. At the same time, the selectivity toward 1,3-propanediol requires the use of catalysts capable of very efficient H_2_ chemisorption and spillover under mild conditions, which limits the choice to the much more expensive noble metals.

### 5.9. Steam Reforming

**Figure 15:**
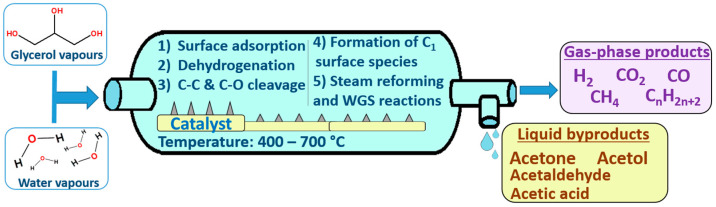
Schematic conceptualization of reaction pathways within the reactor during the glycerol steam reforming process.

Glycerol steam reforming (GSR) can be considered a relatively mature technology compared to the valorization routes described previously, as it aims to convert glycerol into hydrogen and carbon dioxide, thereby positioning glycerol as a potential green energy carrier. The overall GSR reaction can be described by Equation (1), in which one mole of glycerol reacts with three moles of water to produce three moles of CO_2_ and seven moles of H_2_. These final products arise from two consecutive processes: the thermal decomposition of glycerol (Equation (2)) followed by the water–gas shift (WGS) reaction (Equation (3)), with a process sequence similar to the one shown in Figure 15. In addition to these main pathways, GSR involves a complex network of side reactions. These include hydrogen-consuming reactions such as CO and CO_2_ methanation and the reverse water–gas shift (RWGS), as well as carbon-forming reactions like the Boudouard reaction [139], which are among the primary contributors to catalytic coking and deactivation. Beyond these typical reactions, which are also encountered in conventional methane steam reforming, the partial cleavage of glycerol can generate a variety of oxygenated intermediates and liquid byproducts, including acetone, hydroxyacetone (acetol), allylic alcohols, acetaldehyde, and acetic acid, all of which negatively affect the overall hydrogen yield (Figure 15). As can be inferred from the high reaction enthalpy under standard conditions, the overall process is strongly endothermic and thermodynamically demanding. Consequently, GSR is typically conducted at high temperatures, generally in the range of 400–700 °C. Besides temperature, key operational parameters influencing hydrogen yield include system pressure, the water-to-glycerol feed ratio, and the reactant-to-inert gas ratio [62]. Thermodynamic studies have shown that high temperatures, near-atmospheric pressure, low reactant dilution in inert gas, and relatively low gas flow rates are favorable for maximizing hydrogen production [140]. An optimal water-to-glycerol feed ratio has also been identified, typically around WGFR~9.

GSR: C_3_H_8_O_3_ + 3H_2_O → 3CO_2_ + 7H_2_ Δ_r_H = +123 kJ/mol (1)

GD: C_3_H_8_O_3_ → 3CO + 4H_2_ Δ_r_H = +245 kJ/mol (2)

WGS: CO + H_2_O → CO_2_ + H_2_ Δ_r_H = −41 kJ/mol (3)

Catalysts employed for this reaction can generally be described as bifunctional. On one hand, they contain a highly dispersed metallic phase responsible for the cleavage of C-C, C-O, and O-H bonds in glycerol and its reaction intermediates. Noble metals such as Pt, Rh, Ir, and Pd are typically very active, often highly selective toward hydrogen formation, and particularly stable under the harsh high-temperature and oxidative environments characteristic of GSR. They are less prone to sintering and more resistant to deactivation by catalytic coking; however, their large-scale application remains strongly limited by their high cost [141]. Among more affordable transition metals, nickel is the most widely used active phase. Despite its tendency to promote side reactions such as methanation and the reverse water–gas shift (RWGS), Ni exhibits excellent chemisorption properties and strong bond-cleavage capability. Its main limitations are its higher susceptibility to sintering and carbon deposition under reforming conditions [139]. The second key catalytic functionality arises from the support on which the metal nanoparticles are dispersed. A wide variety of supports, such as aluminates, perovskites, phyllosilicates and mixed metal oxides, have been investigated for GSR, many of which provide alkaline surface properties, high surface area, and strong metal–support interactions (SMSI). Beyond stabilizing the metallic phase, these supports also play an important role in water activation, promotion of the water–gas shift reaction, and mitigation of carbon deposition, thereby generating synergistic effects that enhance both catalytic activity and long-term stability [142]. Zhu et al. [141], for instance, investigated an interesting effect associated with strong metal–support interactions (SMSI) in Ni^0^/TiO_2_ anatase catalysts during glycerol steam reforming. In these materials, during the reductive pretreatment step (at 500 °C), the newly formed metallic Ni nanoparticles were found to be encapsulated within a thin anatase TiO_2_ overlayer. Subsequently, under actual GSR operating conditions at 600 °C, this anatase shell underwent a phase transition into rutile TiO_2_, leading to the progressive exposure of catalytically active Ni^0^ particles. The catalyst reduced at 500 °C (Ni/Ti-500), which exhibited this dynamic behavior, initially showed an induction period with glycerol conversion around 45%. However, after approximately 11 h on stream coinciding with the anatase-to-rutile transition, the conversion increased dramatically, reaching nearly 96% and producing about 5.93 mol_H2_/mol_gly_, remaining stable for at least 31 h. In contrast, the catalyst reduced at 600 °C (Ni/Ti-600R), which already possessed a rutile shell, was immediately active and achieved ~98% conversion but suffered rapid deactivation after roughly 20 h due to sintering and carbon deposition. Phyllosilicate nanoscrolls have also been explored as supports for high loadings of Ni, Co, and Ni-Co alloys by Khrapova et al. [142], aiming to highlight synergistic effects not only between support and active metal but also within intermetallic phases. In their study, different Ni/Co molar ratios were supported on sepiolite-derived phyllosilicates ((Co_x_Ni_1−x_)_3_Si_2_O_5_(OH)_4_; x = 0, 0.4, 1). While the Ni-rich sample (x = 0) showed higher initial reactivity due to the greater intrinsic activity and exposure of Ni^0^ sites compared to Co^0^ (x = 1), the intermetallic composition with x = 0.4 displayed a distinctive feature: a progressive increase in glycerol conversion and hydrogen yield over time on stream, outperforming under the stability point of view the monometallic counterparts under identical conditions (Table 13). With the dual aim of ensuring high stability of Ni^0^ nanoparticles while also achieving high glycerol conversion and minimizing the formation of liquid byproducts, Luisetto et al. [139] investigated the benefits of perovskite oxide supports based on CaZrO_3_, SrZrO_3_, and BaZrO_3_ prepared via an autocombustion method (denoted CaZNi-7, SrZNi-7, and BaZNi-7). In their study, the authors demonstrated that the superior catalytic performance of the SrZNi-7 sample was closely related to its support-derived physicochemical properties, including higher surface area, improved nickel reducibility, greater exsolution of Ni from the perovskite matrix, and a higher density of weak basic sites. These features collectively enhanced catalytic behavior compared to the other materials, maximizing hydrogen yield (3.5 mol H_2_ per mol glycerol) while effectively suppressing the formation of liquid byproducts at temperatures above 500 °C. Beyond catalyst design, another important factor influencing process performance lies in reactor configuration. Most of the previously discussed studies rely on so-called traditional reactors (TRs) [143]. However, since glycerol steam reforming is conceptually derived from the more industrially mature methane steam reforming process, it can benefit from more advanced hybrid reactor systems compared to conventional fixed-bed setups. These include sorption-enhanced reactors (SERs), membrane reactors (MRs), and sorption-enhanced membrane reactors (SEMRs). A recent study by S. Macedo et al. [143] systematically compared hydrogen yields obtained under identical operating conditions using these four reactor configurations with a NiAlLaO_x_ catalyst. The results showed that although all systems achieved nearly complete glycerol conversion (notably using diluted crude glycerol as feedstock), the SER configuration integrating an in situ CO_2_ capture component provided approximately 9% higher hydrogen yield than a traditional reactor at 475 °C and 1 bar. An even larger improvement (~15%) was observed with the membrane reactor equipped with a Pd-based hydrogen-permeable membrane, which selectively removed hydrogen from the reaction environment, thereby shifting reaction equilibria and suppressing competing hydrogen-consuming pathways. The combination of both strategies in the SEMR configuration resulted in a total hydrogen yield increase of about 26% relative to a conventional fixed-bed reactor, reaching 6.7 mol H_2_ per mol glycerol, a value approaching the theoretical maximum (Equation (1)).

In conclusion, the strongly endothermic and thermodynamically demanding steam reforming process requires high operating temperatures, high water-to-glycerol feed ratio, the use of bifunctional catalysts containing highly dispersed metallic phase with strong bond-cleavage capability and high-surface-area catalytically active supports that provide alkaline surface properties, favor strong metal–support interactions, promote the water–gas shift reaction and limit carbon deposition. Additionally, the reactor design is of substantial importance, as it could significantly improve the catalytic performance by selectively removing hydrogen from the reaction environment, thus shifting the reaction equilibria and suppressing competing hydrogen-consuming pathways.

## 6. Perspectives, Outlook and Conclusions

Based on the analysis presented throughout this review, the current valorization pathway of glycerol derived from biodiesel waste can be broadly described as being constrained by two major bottlenecks: its purification and its selective conversion into high-value chemicals. The purification of crude glycerol is, in turn, limited by two main challenges. The first is the requirement to achieve very high purity levels in order to meet the standards of the pharmaceutical, food, and cosmetic industries. The second is intrinsically related to the heterogeneous composition of crude glycerol, which introduces significant techno-economic constraints. As discussed in Section 3, the strategies required to obtain high-purity glycerol strongly depend on the initial composition of the crude stream, which itself is influenced by the type of feedstock and the specific biodiesel production process employed. The lack of standardization in crude glycerol streams, therefore, necessitates tailored pretreatment steps aimed at removing a substantial fraction of contaminants, typically enabling purification up to approximately 95%. Further upgrading to the purity levels required by high-value markets necessitates the implementation of advanced purification techniques, often in combination and in a process-specific manner. However, as discussed in Section 4, methods such as vacuum distillation, membrane separation, and ion-exchange processes are not exempt from significant techno-economic limitations, as summarized in Table 3. Although these three purification techniques are capable of achieving high glycerol purities (typically in the range of 97–99%) and high recovery rates from crude streams (approximately 86–99%), they are associated with considerable capital and operating costs. Bansod et al. [18] recently performed a comparative assessment of these technologies, evaluating their performance in terms of productivity, yield, resource consumption, waste generation, energy demand, and maintenance costs. Their study showed that, while ion-exchange and membrane separation processes can achieve high yields and purities, they are characterized by significantly higher fixed capital costs (approximately 6 M$) compared to vacuum distillation (around 4 M$). These higher costs are mainly associated with the substantial operating expenses of the respective technologies. In the case of ion-exchange, costs are largely driven by resin regeneration requirements, whereas membrane separation is penalized by the high solvent consumption needed for membrane treatment and its relatively low energy efficiency. Vacuum distillation, therefore, emerges at present as the most economically viable and robust technology, showing greater resilience to fluctuations in feedstock quality and market conditions compared to the other techniques. The development and improvement of the alternative purification techniques discussed in this review are therefore essential for the design of integrated and hybrid purification systems with lower energy demand and reduced operating costs. Future research should focus more on the development of ion-exchange resins that are less sensitive to variations in crude glycerol composition and capable of being regenerated at moderate cost. At the same time, membrane-based separation technologies will need to evolve toward more fouling-resistant materials that do not require extensive physicochemical pretreatment steps to achieve efficient separation performance.

Despite the progress achieved in purification technologies, the catalytic valorization of glycerol still represents a major bottleneck in the overall value chain. In this review, the most recent strategies and advancements related to the main glycerol conversion pathways toward a wide range of fine chemicals have been thoroughly discussed. However, it is equally important to critically assess the specific limitations associated with each of these processes in order to identify the key challenges that still hinder their large-scale implementation.

Glycerol acetalization can be regarded as a relatively mature and well-established valorization pathway capable of producing commercially relevant oxygenated compounds under mild operating conditions and with high selectivity. Recent advances in catalyst design, particularly the rational tuning of Brønsted–Lewis acidity, the use of porous structured supports, and the implementation of process-intensification strategies such as microwave irradiation, have further improved reaction rates, reduced energy requirements, and enhanced catalytic efficiency. Despite these technological progresses, several limitations remain from a techno-economic and industrial perspective [57]. Chief among them is the intrinsic equilibrium-controlled nature of the reaction, which typically necessitates high acetone-to-glycerol molar ratios to drive acetal formation, leading to additional costs associated with acetone recovery and recycling. Similarly, the continuous removal of the water formed during the reaction is often required to avoid equilibrium back-shifting, adding process complexity. Furthermore, the strong sensitivity of acid catalysts to water and inorganic impurities generally demands glycerol of technical or higher purity, thereby limiting the direct utilization of crude glycerol streams, which commonly contain substantial amounts of residual water, salts, and organic contaminants. Regarding the esterification reaction of glycerol, recent catalyst design strategies have increasingly focused on enhancing porosity, tuning acid site distribution, and improving molecular diffusion in order to overcome the intrinsic selectivity limitations in glycerol acetylation. While high glycerol conversions (often exceeding 80–100%) are readily achieved, particularly under excess acetic acid conditions, the selective formation of fully esterified products remains a major challenge. Most heterogeneous catalytic systems reported in the literature preferentially yield mixtures of mono- and diacetins or exhibit selectivity primarily toward DAG, whereas high triacetin selectivities (>60%) are rarely obtained without resorting to strong mineral acids or acetic anhydride (more expensive than acetic acid). Furthermore, the widespread reliance on excess reactants and continuous water removal continues to raise concerns regarding process scalability and techno-economic feasibility. Very similar criticism was encountered in the etherification reaction of glycerol with alcohols, slightly improved by the etherification reaction using isobutene. However, despite its clear mechanistic advantages and ability to selectively form higher glycerol ethers, isobutene-mediated etherification remains constrained by economic and process-related factors, which currently hinder its large-scale implementation for bulk glycerol upgrading.

Although glycerol dehydration has been widely investigated, and high glycerol conversions and acrolein selectivities can be obtained under optimized laboratory conditions, several intrinsic limitations still restrict the practical applicability of this reaction. The process typically requires high temperatures, strong acid sites, and the presence of water, conditions that inevitably promote catalyst deactivation and limit long-term stability due to carbonaceous deposits. Approaches aimed at improving catalyst stability, such as lowering acid site density or introducing hierarchical porosity, generally result in a compromise between activity, selectivity, and catalyst lifetime. In addition, gas-phase operation at elevated temperatures and the use of dilute aqueous feeds negatively affect process efficiency and space–time yields. As a result, the current limitations of glycerol dehydration are not exclusively associated with catalyst formulation but also with inherent reaction and process constraints. Overcoming these challenges will likely require advances beyond catalyst optimization alone, including improved reactor design, regeneration strategies, or alternative reaction pathways.

As already extensively described, the glycerol oligomerization reaction presents potential but also serious limitations. Despite the wide variety of catalytic systems investigated, ranging from homogeneous bases and ionic liquids to heterogeneous LDHs, mixed oxides, and modified natural minerals, most catalysts ultimately lead to similar product distributions, typically consisting of mixtures of diglycerols, triglycerols, and higher oligomers. Achieving strict selectivity toward a single oligomer class remains challenging, since improved control over polymer growth often comes at the expense of catalytic activity, as observed in balanced acid–base systems. Another common feature, especially for hetero-catalyzed reactions, is the relatively moderate glycerol conversion, which rarely exceeds 60–70% under typical conditions and tends to reach a plateau over time due to equilibrium limitations, increased viscosity of the reaction medium, and progressive diffusion constraints. Additionally, the reaction requires relatively high temperatures, generally above 220 °C, to proceed at practical rates, which further promotes side reactions and accelerates catalyst deactivation. As a result, while glycerol oligomerization represents a promising route for producing value-added chemicals, its practical implementation remains constrained by fundamental challenges related to selectivity control, catalyst durability, and process operability.

The direct carbonation of glycerol with CO_2_ represents the most conceptually attractive route from a sustainability standpoint, as it couples two abundant waste streams into a single high-value product. However, this pathway remains intrinsically limited by unfavorable thermodynamics, equilibrium constraints due to water formation, and the need for high CO_2_ pressures and sacrificial dehydrating agents. These factors significantly complicate the process design and reduce its practical feasibility at scale. More broadly, when considering all glycerol carbonation strategies, including transesterification with organic carbonates and urea-based routes, common limitations emerge. These include the frequent requirement for excess reagents to shift equilibrium, catalyst deactivation or leaching over repeated cycles, sensitivity to impurities in crude glycerol, and downstream separation challenges that can negatively impact overall process economics. Thus, while significant catalytic advances have enabled high conversions and selectivities under optimized laboratory conditions, further progress is still needed to reconcile catalytic performance with realistic feedstocks, energy efficiency, and scalable process integration.

Glycerol oxidation would enable the selective production of a wide spectrum of oxygenated chemicals through thermocatalytic, electrocatalytic and photocatalytic approaches. While thermocatalysis benefits from relatively high conversion levels and well-established reactor concepts, it often requires pressurized oxidants and carefully tuned acid–base environments to control selectivity. Electrocatalytic oxidation, on the other hand, operates under milder conditions and offers the additional advantage of coupling chemical production with hydrogen generation, yet it frequently suffers from low glycerol conversion and relies mainly on Faradaic efficiency as a performance metric. In both cases, catalyst stability, product separation, and process scalability remain key challenges. Photocatalytic glycerol oxidation, although attractive, remains limited by low quantum efficiency and fast electron–hole recombination. Selectivity is also difficult to control due to overoxidation and C-C cleavage rather than the formation of desired products. Enhanced performances frequently rely on complex catalyst architectures, raising concerns about reproducibility and practical application. Finally, scalability remains a key challenge compared to thermocatalytic and electrocatalytic routes, mainly due to limitations in light penetration, reactor design, and overall energy efficiency. Despite significant progress at the laboratory scale, further development is required to translate glycerol oxidation into an economically competitive industrial technology.

Hydrogenolysis of glycerol is one of the most attractive valorization routes, mainly because it operates under relatively mild aqueous conditions, tolerates crude glycerol feeds, and can be coupled with in situ hydrogen generation via APR and water–gas shift reactions. In addition, both target products, 1,2- and 1,3-propanediol, are valuable bulk chemicals with established markets. However, the two pathways differ significantly in feasibility. The synthesis of 1,2-propanediol is comparatively straightforward, as it is both kinetically and thermodynamically favored. Efficient selectivities can be achieved using relatively inexpensive Ni- and Cu-based catalysts that promote C-O cleavage while limiting C-C scission. Still, challenges remain, including catalyst deactivation, competing side reactions under harsh conditions, and hydrogen management when relying on autogenous production. In contrast, selective formation of 1,3-propanediol is intrinsically more difficult due to the lower reactivity of the secondary hydroxyl group. High selectivity typically requires low temperatures, high hydrogen pressures, and bifunctional catalysts with strong metal–support interactions, most often based on noble metals. This reliance on costly materials, together with long reaction times and limited studies under continuous or self-sustained hydrogen conditions, continues to constrain the practical scalability of this route.

Steam reforming represents one of the most technologically mature routes for glycerol valorization, offering the attractive possibility of converting an abundant bio-derived waste into hydrogen-rich gas streams. Significant progress has been achieved in catalyst design, particularly through the development of bifunctional systems that combine efficient C-C/C-O bond cleavage activity with improved resistance to sintering and carbon deposition. Advances in support engineering, metal–support interactions, and reactor configurations such as sorption-enhanced and membrane-assisted systems have further demonstrated that hydrogen yields can approach theoretical limits under optimized conditions. Nevertheless, the process still faces intrinsic challenges related to its highly endothermic nature, the need for elevated operating temperatures, and the persistence of competing side reactions that lower hydrogen selectivity and promote catalyst deactivation over time. In this context, future improvements will likely depend on balancing catalytic stability, energy efficiency, and process integration, particularly through the combined optimization of catalyst formulations and intensified reactor designs.

A recurring theme across all these pathways is the strong dependence of catalytic performance on feed purity, catalyst stability, and process conditions. Catalyst deactivation (via sintering, coking, or leaching), mass transfer limitations, and the formation of complex product mixtures remain key bottlenecks. Moreover, many reported systems still rely on noble metals or exhibit limited long-term stability, raising concerns regarding economic feasibility at scale. In addition, for pathways yielding high-value products with potential pharmaceutical relevance, such as oxidation to dihydroxyacetone or glyceric acid, acetalization to solketal, esterification to glycerol-derived excipients, and carbonation to glycerol carbonate, an important yet underexplored issue is the possible contamination of products by leached metal species. While metal leaching is often discussed in relation to catalyst stability and recyclability, its impact on product purity is not addressed. This aspect is particularly critical for pharmaceutical applications, where strict limits on residual metals apply. However, the current literature provides limited quantitative data on metal leaching and its transfer to the product phase. Bridging this gap between catalytic performance and downstream purity requirements remains a key challenge, warranting more systematic investigation in future studies. Another limitation identified in this study relates to the scarcity of catalytic examples in the various value-added pathways using refined crude glycerol (approximately 95% purity) or even in the direct use of crude glycerol. The majority of studies were found to use technical-grade glycerol to validate their results. The scientific community must make a greater effort to apply the catalytic results obtained from glycerol feedstocks with higher impurity levels in order to minimize the demand for purification.

In conclusion, while glycerol represents a highly versatile and abundant platform molecule, its effective utilization requires a holistic approach that bridges purification, catalysis, and process engineering. Continued efforts in these areas will be crucial to unlocking the full potential of glycerol within future circular and sustainable chemical industries.

## Figures and Tables

**Table 1:** Fatty acid composition (wt.%) of selected biodiesel feedstocks: vegetable oils, oil blends, and used cooking oil (UCO).

Type of FFA	C Chain	[10]	[11]	[12]	[13]	[13]	[14]
Oils Source	-	Blend	UCO	Jatropha	Rapeseed	Sunflower	Palm
Myristic acid	C14:0	1	-	-	-	-	0.8
Palmitic acid	C16:0	44	10.2	13	7.2	4.3	40.8
Palmoleic acid	C16:1	-	2.4	-	0.06	-	0.1
Stearic acid	C18:0	4.5	44.2	8.6	5.3	2.6	5.2
Oleic acid	C18:1	39	35.6	45.4	18.5	66.3	35.7
Linoleic acid	C18:2	9.5	3.5	33	66.3	18.6	9.3
Linolenic acid	C18:3	0.4	-	-	0.09	6.9	0.2
Others	-	1.4	4.0	-	2.55	1.32	7.9

**Table 2:** Crude glycerol composition from different biodiesel feedstocks.

Parameters	[19]	[28]	[30]	[31]	[29]
Oil crude Derivation	-	Soybean	Jatropha	Sunflower	Palm biomass
Glycerol content (wt.%)	35.60	33.3	37.1	63.2	81.7
Ash content (wt.%)	4.73	2.8	3.7	2.1	9.4
Methanol content (wt.%)	N.D.	12.6	1.2	34.4	N.D.
Water content (wt.%)	9.38	6.5	19.5	1.6	14.1
MONG content (wt.%)	50.29	48.4	30.50	N.D.	9.0
pH	9.6	9.5	8.6	13.1	7.4
Color	Dark Brown	N.D.	N.D.	N.D.	Light Brown

**Table 3:** Comparison of advantages and disadvantages of different techniques for crude glycerol purification.

Technique	Advantages	Disadvantages	Refs.
Chem-Phys methods	– Generally is a necessary pretreatment – Low-energy requirements – Large-scale flexibility – Removes MONGs	– Limited glycerol purity achieved – Partial removal of salts – Multistep process – Requires solvents and evaporators	[32,33]
VD	– Established Technique – Produce high-purity glycerol – Large-scale production	– Energy Intensive – High maintenance costs – Impurities limit yields	[18,37]
MP	– Economically feasible and Scalable – Medium rate flux – Selective for particles, macromolecules and soaps	– MONGs cause membrane fouling – Pretreatment step is necessary – Membrane solvent washing is needed – Limited glycerol purity achieved	[41,42]
MD	– Produce high-purity glycerol – Low rate flux – Moderate temperatures – Moderate pressures	– Significant heat loss – Impurities and wetting of the membrane cause fouling – Still not widely used on an industrial scale	[36,45]
ED	– Effective removal of ions – Low energy consumption – Reduced use of chemical reagents – Continuous-flow process	– Pretreatment step is necessary – Possible fouling of the membranes – Limited effectiveness on non-ionic compounds and high salt contents – Sensitive to operating conditions	[49,50]
IE	– Effective removal of ions – High purity achieved – Operational flexibility provided by different resin combinations – Moderate operating conditions	– Regeneration of resins is necessary – Significant cost of resins – Possible organic fouling of the resins – Resin pretreatment step is needed	[51,52]
AD	– Decolorization – Removal of odors – Large-scale flexibility	– Requires secondary filtration processes – Recycling of the adsorbent is not guaranteed	[53,56]

**Table 4:** Recent literature results on the catalytic conversion of glycerol to solketal.

Catalyst	Reaction Conditions	Main Product	Glycerol Conv. (%)	Product Sel. (%)	Ref.
20–MoO_3_-ZrO_2_	– Temperature: 50 °C – Acetone/Glycerol ratio: 8 – Reaction time: 10 min – Catalyst amount: 30 mg	Solketal	89	97	[68] 2024
SA-60	– Temperature: 60 °C – Acetone/Glycerol ratio: 10 – Reaction time: 1.5 h – Catalyst amount: 80 mg	Solketal	94.6	97	[69] 2026
SBA-SO_3_H	– Microwaves used – Power: 50–100 W – Temperature: 40 °C – Acetone/Glycerol ratio: 12 – Reaction time: 2 min – Catalyst amount: 5 wt.%	Solketal	90	97	[66] 2022
RhMOP-SO_3_H	– Temperature: 60 °C – Acetone/Glycerol ratio: 4 – Reaction time: 1 h – Catalyst amount: 0.1 wt.%	Solketal	86	97	[70] 2025
BP–SO_3_H-15-18-100	– Microwaves used – Temperature: 65 °C – Acetone/Glycerol ratio: 4 – Reaction time: 12 min – Catalyst amount: 7 wt.%	Solketal	94.9	97.5	[71] 2024
Zr-S-400	– Temperature: 40 °C – Acetone/Glycerol ratio: 6 – Reaction time: 1 h – Catalyst amount: 0.6 wt.%	Solketal	80	86	[72] 2021

**Table 5:** Selected examples of recent literature results on the catalytic oxidation of glycerol.

Catalytic Reaction	Catalyst	Metal Loading (wt.%)	Reaction Conditions	Main Product	Glycerol Conv. (%)	Product Sel. (%)	Ref.
Thermo-	Au-Pt/Mn_x_O_y_C_z_	3	– 0.1 M Gly. – Alkaline – 60 °C – 1 MPa O_2_ – 2 h	Glyceric Acid	100	57.3	[75] 2025
Thermo-	Au/Cu_0.1_Zn_0.9_O-MR	1	– 0.1 M Gly. – Neutral – 100 °C – 1 MPa O_2_ – 2 h	Dihydroxyacetone	100	82.3	[76] 2026
Electro-	10Pt/Au/Ti	ALED	– 0.1 M Gly – Alkaline – 0.1 M KOH – 0.9 V_RHE_ – No O_2_ used – 2 h	Glyceric Acid	14.6	93.6	[78] 2026
Electro-	0.1Cu-Ni_3_S_2_@NF	1:10 Cu:Ni	– 0.5 M Gly – Alkaline – 0.1 M KOH – 100 mA/cm^2^ – 1.46 V_RHE_ – No O_2_ used – 10 h	Formic Acid	~30	-	[79] 2024
Photo-	Co-TiO_2_ (anatase)	0.4	– 1 M Gly – 100 μL vol. – 365 nm – 10 h	Formic Acid	95	57	[81] 2025
Photo-	AKCN	-	– 0.01 M Gly. – 20 mL vol. – 300 W Xenon – 6 h – O_2_ used	Glyceric Acid & Dihydroxyacetone	~80	~70	[82] 2024
Photo-	TeKCNIr	10	– 0.01 M Gly. – 20 mL vol. – 450 nm – O_2_ used	Glyceraldehyde	~45	88	[83] 2024

**Table 6:** Recent catalytic results for glycerol esterification with acetic acid.

Catalyst	Glycerol/AcOOH	Temperature (°C)	Time (h)	Glycerol Conv. (%)	Selectivity (%)			Ref.
					MAG	DAG	TAG	
Sn_3/2_PW_12_O_40_	1:3	60	8	65	15	74	11	[87] 2025
20%Cs/DTP/K10	1:9	120	4	92.5	46	48	6	[89] 2025
P-20PA-KIT-6	1:9	115	8	100	6.7	41.3	52.0	[90] 2025
H-ZSM-5	1:9	115	8	99	16.9	59.4	24	[90] 2025
Amberlyst-15	1:9	115	8	99	15.7	50.7	34	[90] 2025
MOF-808	1:9	100	4	62.8	66.9	33.1	0	[91] 2025
MOF-808-PLU	1:9	100	4	71.6	5.6	84.1	10.4	[91] 2025
Ce/TiZrO_4_@SO_4_^2−^	1:6	120	1	36	2	96	2	[93] 2025
10SA	1:6	100	2	100	8	51	41	[94] 2021
30NiO/TiO_2_	1:10	170	0.5	90	20	15	66	[95] 2021

**Table 7:** Recent catalytic results for glycerol etherification with different alcohols.

Sample	ROH *	Gly/ ROH	Temperature (°C)	Time (h)	Glycerol Conv. (%)	Selectivity (%)			Ref.
						MGE	DGE	TGE	
Amberlyst-15	EtOH	1:12	110	6	97.5	19.5	37.6	42.8	[51] 2023
Amberlyst-15	iPrOH	1:12	110	6	85.2	97.1	2.8	0	[51] 2023
CeHBeta	tBuOH	1:4	90	2	81.6	86.4	2.7	13.5	[98] 2026
PSK-160	tBuOH	1:4	90	2	50.7	83.1	10.8	6.04	[99] 2025
HSn_1.5_SiW_12_O_40_	tBuOH	1:4	100	4	65	63	19	-	[60] 2022
Sn_1.5_PMO_12_O_40_	tBuOH	1:4	100	4	67	65	18	-	[60] 2022

* ROH: EtOH = ethanol, iPrOH = iso-propanol, tBuOH = tert-butanol.

**Table 8:** Recent catalytic results for glycerol isobutene-mediated etherification; “auto.” refers to a system under autogenous pressure.

Sample	Gly:IB:tBuOH	Press. (Bar)	Temp. (°C)	Time (h)	Glycerol Conv. (%)	Yield or Select. (%)			Ref.
						MGE	DGE	TGE	
NKC-9	1:4:0	30	90	24	99.8	80 Yield DGE + TGE			[97] 2021
NKC-9	1:0:4	30	90	24	99.8	6 Yield DGE + TGE			[97] 2021
NKC-9	1:3:1	30	90	24	92.0	55 Yield DGE + TGE			[97] 2021
Amberlyst-15	1:3:0	auto.	75	6	100	11.1	58.6	16.3	[100] 2020
Amberlyst-36	1:3:0	auto.	75	6	100	9.2	57.9	16.9	[100] 2020
Zeolite -Y	1:3:0	auto.	75	6	90	28.9	60.1	6.1	[100] 2020
Zeolite Beta	1:3:0	auto.	75	6	100	11.3	73.8	0.8	[100] 2020
TSA	1:3:0	auto.	75	6	78	21.4	47.3	24.1	[100] 2020
TPA	1:3:0	auto.	75	6	85	18.3	47.0	25.0	[100] 2020

**Table 9:** Recent catalytic results for glycerol dehydration to acrolein.

Catalyst	Reaction Conditions	Main Product	Glycerol Conv. (%)	Product Selectivity (%)			Ref.
				Acrolein	Acetol	Propanal	
FePO_4_-HT600	– Temperature: 300 °C – Glycerol_(aq.)_ 10 wt.% – 4.2 g/h feed flow rate – N_2_ carrier flow 5 mL/min – 800 mg catalyst loading – 10 h of experiment	Acrolein	100	93.2	0.07	-	[103] 2025
BPO_4_-1000	– Temperature: 320 °C – Glycerol_(aq.)_ 20 wt.% – 0.5 mL/h feed flow rate – N_2_ carrier flow 2 mL/min – 1 g catalyst loading – 2 h experiment – WHSV: 0.11 h^−1^ – 425 h stability test	Acrolein	100	80	14	-	[102] 2024
F-Al_2_O_3_	– Temperature: 350 °C – Glycerol_(aq.)_ 40 wt.% – LHSV: 1.50 h^−1^ – N_2_ carrier flow 40 mL/min – ~10 g catalyst loading – 2 h experiment	Acrolein	100	79.7	11.1	6.8	[101] 2025
Nb_2_O_5_	– Temperature: 350 °C – Glycerol_(aq.)_ 20 wt.% – LHSV: 1.12 h^−1^ – N_2_ carrier flow 40 mL/min – ~10 g catalyst loading – 2 h experiment	Acrolein	99	80.8	5.7	8.7	[104] 2025
H-ZSM-5	– Temperature: 380 °C – Glycerol_(aq.)_ 40 wt.% – LHSV: 0.37 h^−1^ – N_2_ carrier flow 40 mL/min – ~10 g catalyst loading – 2 h experiment	Acrolein	100	62.2	9.1	8.8	[104] 2025
US-HY	– Temperature: 350 °C – Glycerol_(aq.)_ 40 wt.% – LHSV: 1.50 h^−1^ – N_2_ carrier flow 40 mL/min – ~10 g catalyst loading – 2 h experiment	Acrolein	93	69.1	10.1	4.6	[105] 2025
AT-Z70	– Temperature: 320 °C – Glycerol_(aq.)_ 20 wt.% – WHSV: 1.25 h^−1^ – N_2_ carrier flow 20 mL/min – 1 g catalyst loading – 12 h experiment	Acrolein	97	70	-	-	[105] 2025

**Table 10:** Recent catalytic results for glycerol oligomerization.

Catalyst	Reaction Conditions	Glycerol Conv. (%)	Product Selectivity (%)			Ref.
			Dimers	Trimers	Oligomers	
MAU05P	– Temperature: 240 °C – Time: 8 h – 50 g glycerol volume – 4 wt.% of catalyst – N_2_ atmosphere used	64	36.7	5.7	57.6	[110] 2022
MgAl MO	– Temperature: 240 °C – Time: 8 h – 50 g glycerol volume – 4 wt.% of catalyst – N_2_ atmosphere used	56.9	23.4	7	69.6	[107] 2026
Co/MgAl LDH	– Temperature: 240 °C – Time: 8 h – 50 g glycerol volume – 4 wt.% of catalyst – N_2_ atmosphere used	68.2	24.1	9	66.9	[107] 2026
Dolomite	– Temperature: 220 °C – Time: 24 h – 50 g glycerol volume – 3 wt.% of catalyst – N_2_ atmosphere used	76.7	52.5	6.4	41.2	[111] 2022
Mg_1_Al_1_/LDO/ CaCO_3_	– Temperature: 220 °C – Time: 24 h – 50 g glycerol volume – 3 wt.% of catalyst – N_2_ atmosphere used	52.1	92	7.4	0.6	[111] 2022
K_2_ZnF_6_	– Temperature: 230 °C – Time: 18 h – 50 g glycerol volume – 4 wt.% of catalyst – N_2_ atmosphere used	68	27	9	64	[108] 2024

**Table 12:** Recent catalytic results achieved from the hydrogenolysis of glycerol to 1,2- and 1,3-propandiol. Product selectivity refers to the main product.

Catalyst	Reaction Conditions	Main Product	Glycerol Conv. (%)	Product Sel. (%)	Ref.
Cu_35_Fe_18_Zn_45_Al_2_– HDT	– Batch conditions – Temperature: 230 °C – Time: 6 h – H_2_ pressure: 4 MPa – Glycerol_(aq)_: 30 wt.% – Catalyst loading: 12.5 wt.%	1,2-PD	100	96.4	[132] 2026
CuAl-400 (DC)	– Batch conditions – Temperature: 220 °C – Time: 5 h – H_2_ pressure: 5.2 MPa – Glycerol_(aq)_: 20 wt.% – Catalyst loading: 1 wt.%	1,2-PD	60	94	[128] 2025
1B-NiAl	– Flow conditions – Temperature: 235 °C – Back pressure: 45 bar – WHSV: 12.2 h^−1^ – H_2_ autogenous – Glycerol_(aq)_: 10 wt.% – Catalyst loading: 0.5 g	1,2-PD	86	30.2	[133] 2025
1V-NiAl	– Flow conditions – Temperature: 235 °C – Back pressure: 45 bar – WHSV: 12.2 h^−1^ – H_2_ autogenous – Glycerol_(aq)_: 10 wt.% – Catalyst loading: 0.5 g	1,2-PD	82.5	40.7	[133] 2025
NiCeZr-0.15	– Flow conditions – Temperature: 235 °C – Back pressure: 35 bar – WHSV: 12 h^−1^ – H_2_ autogenous – Glycerol_(aq)_: 10 wt.% – Catalyst loading: 0.5 g	1,2-PD	75.3	52.8	[127] 2024
Cu-Ni/30MgO-Al_2_O_3_	– Flow conditions – Temperature: 235 °C – Back pressure: 40 bar – WHSV: 2 h^−1^ – Flow rate: 0.04 mL/min – H_2_ autogenous – Glycerol_(aq)_: 10 wt.% – Catalyst loading: 1.25 g	1,2-PD	80	50	[134] 2022
Pt/W-SiO_2_-700	– Batch conditions – Temperature: 140 °C – Time: 12 h – Hydrogen pressure: 5 MPa – Glycerol_(aq)_: 5 wt.% – Catalyst loading: 100 mg – Pt loading: 3.8 wt.%	1,3-PD	70.9	61.3	[135] 2025
Pt-WO*ₓ*/RTNR-453	– Batch conditions – Temperature: 150 °C – Time: 24 h – Hydrogen pressure: 4 MPa – Glycerol_(aq)_: 30 wt.% – Catalyst loading: 0.4 g – Pt loading: 2.9 wt.%	1,3-PD	96.7	60.6	[136] 2026
4Pt-0.6W-0.05Ir/G-6	– Batch conditions – Temperature: 140 °C – Time: 8 h – Hydrogen pressure: 6 MPa – Glycerol_(aq)_: 5 wt.% – Catalyst loading: 200 mg	1,3-PD	50	56	[137] 2025
IrRe-H + Amberlyst-15	– Batch conditions – Temperature: 120 °C – Time: 8.5 h – Hydrogen pressure: 8 MPa – Glycerol_(aq)_: 20 wt.% – Catalyst loading: 400 mg	1,3-PD	65.2	45.7	[138] 2025

**Table 13:** Recent catalytic results achieved by steam reforming of glycerol.

Catalyst	Reaction Conditions	Glycerol Conv. (%)	Product Selectivity (%)				Ref.
			H_2_	CH_4_	CO	CO_2_	
Ni/Ti-500R	– Temperature: 600 °C – WHSV = 9 h^−1^ – Glycerol_(aq)_: 30 wt.% – Feed rate: 0.03 mL/min – N_2_/feed ratio: 4 – Catalyst amount: 200 mg – Ni loading: 10.4 wt.% – Stability: 21 h	80	~95	<0.5	~10	~85	[141] 2024
(Co*_x_*Ni_1-*x*_)_3_Si_2_O_5_(OH)_4_; x = 0.4	– Temperature: 600 °C – GHSV = 80,000 h^−1^ – Glycerol_(aq)_: 20 wt.% – Feed rate: 7.4 mL/h – N_2_/feed ratio: 243 – Catalyst amount: 120 mg – Ni-Co loading: ~52 wt.% – Stability: 4 h	70–80	60	4	67	29	[142] 2025
SrZNi-7	– Temperature: 600 °C – WHSV = 50,000 mL g^−1^ h^−1^ – Glycerol_(aq)_: 31 wt.% – Feed rate: 0.12 mL/min – He/feed ratio: 416 – Catalyst amount: 200 mg – Ni loading: 9.3 wt.% – Stability: 20 h	85	~95	~5	~20	~75	[139] 2025
Ni/CeO_2_-PC	– Temperature: 500 °C – GHSV = 28,500 mL g^−1^ h^−1^ – Glycerol_(aq)_/water: 9 – Feed rate: 0.02 mL/min – Catalyst amount: 200 mg – Ni loading: 7 wt.% – Stability: 25 h	90	92	12	8	80	[144] 2024
15CSZ11	– Temperature: 700 °C – Glycerol_(aq)_: 30 wt.% – Feed rate: 0.08 mL/min – N_2_/feed ratio: 125 – Catalyst amount: 1 g – Co loading: 15 wt.% – Stability: 50 h	100	72	3.8	18.2	78	[62] 2022

## Data Availability

Not applicable.

## Abbreviations

The following abbreviations are used in this manuscript:

1,2-PD	1,2-propanediol	MGE	monoalkyl glycerol ethers
1,3-PD	1,3-propanediol	MONG	Matter Organic Non-Glycerol
AD	Adsorption	MP	Membrane Purification
ALED	atomic layer electrodeposition	MTBG	mono-tert-butyl glycerol ether
APH	aqueous phase hydrogenolysis	MWCO	molecular weight cut-off
CEMD	Continuous-Effect Membrane Distillation	NF	nanofiltration
DAG	diacetin	OEC	Observatory of Economic Complexity
DCMD	Direct Contact Membrane Distillation	OER	oxygen evolution reaction
DGE	dialkyl glycerol ethers	PFAD	palm fatty acid distillates
DMC	dimethyl carbonate	PLU	Pluronic P123
EBB	European Biodiesel Board	POME	palm oil mill effluent
ED	Electrodialysis	RSM	response surface methodology
EFSA	European Food Safety Authority	RWGS	reverse water-gas shift
FAME	fatty acid methyl esters	SEMR	sorption-enhanced membrane reactors
FCC	Food Chemicals Code	SER	sorption-enhanced reactors
FFA	free fatty acids	SMSI	strong metal–support interactions
GC	glycerol carbonate	TAG	triacylglycerides or triglycerides
GOR	gain–output ratio	TPA	phosphotungstic acid
GSR	Glycerol steam reforming	TMP	transmembrane pressure
HDTs	hydrotalcite-derived catalysts	TGE	trialkyl glycerol ethers
HEFA	hydrotreated esters and fatty acids	UCO	used cooking oil
HER	hydrogen evolution reaction	UF	ultrafiltration
HPAs	heteropolyacid	USP	United States Pharmacopeia
HVO	hydrotreated vegetable oils	VD	Vacuum Distillation
IE	Ion exchange	WGS	water–gas shift reaction
ILUC	indirect land use change		
LDH	layered double hydroxides		
LDO	layered double oxides		
LHSV	liquid hourly space velocity		
LMBs	lithium metal batteries		
MgAl MO	Mg-Al mixed oxides		
MAG	monoacetin		
MD	Membrane Distillation		
MF	microfiltration		
